# Mediterranean lifestyle index and 24-h systolic blood pressure and heart rate in community-dwelling older adults

**DOI:** 10.1007/s11357-023-00898-z

**Published:** 2023-08-10

**Authors:** Irene Talavera-Rodríguez, José R. Banegas, Juan J. de la Cruz, David Martínez-Gómez, Miguel Ruiz-Canela, Rosario Ortolá, Maria S. Hershey, Fernando Rodríguez Artalejo, Mercedes Sotos-Prieto

**Affiliations:** 1 Department of Preventive Medicine and Public Health, School of Medicine, Universidad Autónoma de Madrid, 28049 Madrid, Spain; 2 CIBERESP (CIBER of Epidemiology and Public Health), Madrid, Spain; 3 IMDEA-Food Institute, CEI UAM + CSIC, Madrid, Spain; 4 Department of Preventive Medicine and Public Health, Navarra Institute for Health Research (IdisNa), University of Navarra, 31008 Pamplona, Spain; 5 CIBEROBN (CIBER of Pathophysiology of Obesity and Nutrition), Madrid, Spain; 6 Department of Environmental Health, Harvard T.H. Chan School of Public Health, 677 Huntington Avenue, Boston, MA 02115 USA

**Keywords:** Ambulatory blood pressure monitoring, 24-h heart rate, Mediterranean lifestyle, Older adult

## Abstract

Specific foods, nutrients, dietary patterns, and physical activity are associated with lower blood pressure (BP) and heart rate (HR), but little is known about the joint effect of lifestyle factors captured in a multidimensional score. We assessed the association of a validated Mediterranean-lifestyle (MEDLIFE) index with 24-h-ambulatory BP and HR in everyday life among community-living older adults. Data were taken from 2,184 individuals (51% females, mean age: 71.4 years) from the Seniors-ENRICA-2 cohort. The MEDLIFE index consisted of 29 items arranged in three blocks: 1) Food consumption; 2) Dietary habits; and 3) Physical activity, rest, and conviviality. A higher MEDLIFE score (0–29 points) represented a better Mediterranean lifestyle adherence. 24-h-ambulatory BP and HR were obtained with validated oscillometric devices. Analyses were performed with linear regression adjusted for the main confounders. The MEDLIFE-highest quintile (vs Q1) was associated with lower nighttime systolic BP (SBP) (-3.17 mmHg [95% CI: -5.25, -1.08]; p-trend = 0.011), greater nocturnal-SBP fall (1.67% [0.51, 2.83]; p-trend = 0.052), and lower HR (-2.04 bpm [daytime], -2.33 bpm [nighttime], and -1.93 bpm [24-h]; all *p*-trend < 0.001). Results were similar for each of the three blocks of MEDLIFE and by hypertension status (yes/no). Among older adults, higher adherence to MEDLIFE was associated with lower nighttime SBP, greater nocturnal-SBP fall, and lower HR in their everyday life. These results suggest a synergistic BP-related protection from the components of the Mediterranean lifestyle. Future studies should determine whether these results replicate in older adults from other Mediterranean and non-Mediterranean countries.

**Supplementary Information:**

The online version contains supplementary material available at 10.1007/s11357-023-00898-z.

## Introduction

Hypertension is a major preventable cause of cardiovascular disease (CVD) and all-cause death worldwide [1], yet remains poorly controlled in many countries and clinical settings [2]. Currently, about 60% of the population 60 + years and 75% of those 75 + years are hypertensive. High blood pressure (BP) is a major risk factor for dementia, and heart and renal failure [3] and in patients aged 50 + years, systolic BP (SBP) is a better predictor of CVD events than diastolic BP [4, 5]. In addition, ambulatory BP monitoring (ABPM) in everyday life and self-measured BP monitoring (SBPM)—the most accurate and comprehensive ways to measure BP- are stronger predictors of CVD and total mortality than office BP [6, 7], with the added advantage of ABPM of measuring BP during the night and nonetheless being well accepted by patients, including older people [8]. Additionally, heart rate (HR) is associated with atherosclerosis and functional decline in older adults [9] and, together with nighttime-SBP and nocturnal-SBP dipping, predict CVD events [10].

Among the main causes of the lack of BP control is the insufficient therapeutic adherence, both pharmacological and non-pharmacological [4, 11], the latter being key for preventing hypertension and the management of high BP in adults [4, 6]. The effect of lifestyle factors on BP has been usually evaluated separately [12], with a few studies on the role of diet on ambulatory BP [13–15]. For example, the Dietary Approach to Stop Hypertension (DASH) and the Mediterranean Diet (MedDiet), are the dietary patterns that have shown the greatest protective effect on BP [16, 17]. Most studies have analysed the effect of DASH on office BP and, less frequently, on ABPM [18, 19] and, to our knowledge, only one study has evaluated the effect of MedDiet on ambulatory BP [20]. Evidence regarding the effect of lifestyle interventions on ambulatory HR is scarce [21] and even less evidence is available on the joint effect of multiple lifestyle factors on ambulatory BP [22] or HR, especially in older adults, where hypertension is more frequent and challenging to control.

To take into account the cultural and social factors related to lifestyle and their potential synergisms, Sotos-Prieto et al. developed the validated multidimensional Mediterranean Lifestyle (MEDLIFE) index, which includes the Mediterranean diet, dietary habits, physical activity, rest, and conviviality [23, 24], and has been associated with lower risk of CVD, frailty, mortality, and other adverse health outcomes in Mediterranean and non-Mediterranean populations [25–30]. While previous studies have assessed the association between a Mediterranean lifestyle and risk of hypertension in 92 firefighters recruits [31] and with lower prevalence of metabolic syndrome in 249 US career firefighters [29]; none have evaluated as main outcome 24 h ambulatory blood pressure nor have included a wider population of community living older adults.

We hypothesized that higher adherence to the MEDLIFE index (as an overall measure of a Mediterranean way of living) is associated with better outcomes on 24 h ABPM. This is the first study to evaluate the association of MEDLIFE index with 24-h SBP and HR in everyday life of older adults.

## Methods

### Study design and population

The Seniors-ENRICA-2 study (trial code: ClinicalTrials.gov number, NCT03541135) is a prospective cohort including 3,273 community dwelling-individuals aged 65 + years. Participants were recruited between 2015 and 2017 by stratified random sampling of individuals holding a national identity card and living in the city of Madrid (Spain) and four surrounding large towns. All people residing in Spain are entitled to free healthcare, so the list of card-holders closely reflects the entire resident population. Information was collected using similar methods and instruments as in the Seniors-ENRICA-1 cohort. [32] Briefly, data were collected in three sequential stages. First, a phone interview by trained staff on sociodemographic, lifestyle, health status, morbidity, and healthcare services use. Second, a home visit by nurses to collect blood and urine samples. Lastly, a second home visit by trained lay personnel to obtain a diet history and to perform a physical exam. The Clinical Research Ethics Committee of *La Paz* University Hospital in Madrid approved the study, and participants gave written informed consent.

### Mediterranean lifestyle (MEDLIFE) index

Habitual food consumption in the preceding year was obtained with HD-ENRICA, a validated electronic face-to-face diet-history [33]. Physical activity was ascertained using the validated EPIC-Spain cohort questionnaire [34]. Leisure activities included walking, cycling and other forms of exercise, as well as gardening, household chores and do-it-yourself activities. Sedentary behaviour was estimated as time spent watching TV, using the computer, reading, commuting, and listening to music, using the Nurses’ Health Study questionnaire validated in Spain [35]. Data on sleep, naptime, conviviality, and dietary habits was self-reported.

The Mediterranean lifestyle (MEDLIFE) index was computed based on the version published and validated by Sotos-Prieto et al. [23, 24] with a few modifications to adapt to the Seniors-ENRICA-1 cohort [28]. Specifically, three new items were added: a) item 17: low sodium consumption; b) item 21: coffee or tea consumption *in lieu of* the original question of water consumption, since this information was difficult to assess; and c) item 29: socializing with friends or family. Also, one item was removed (cereals) since item 18 already records the preference for whole grain foods. Other minor modifications include changes in cut-off criteria of nuts, fruits, and wine consumption (Appendix Table 1).

This modified MEDLIFE consists of 29 items divided into three blocks describing: 1) Food consumption (14 items); 2) Dietary habits (8 items); and 3) Physical activity, rest, and conviviality (7 items). Each item was scored 0 points, if the criterion was not met, and 1 point, if met (Appendix Table 1). Therefore, the index ranges from 0 (worst) to 29 (best adherence to Mediterranean lifestyle). Scores were categorized into quintiles (Q1: lowest adherence).

### Outcome

24-h ambulatory BP and HR were measured with a validated oscillometric device (Mobil-O-Graph 24 h PWA monitor, I.E.M., Stolberg, Germany) and appropriate size cuffs placed on the non-dominant arm [36]. The device registered BP at 20-min intervals during the day and 30-min intervals at night. Readings were conducted preferably on working days. Daytime and nocturnal periods, defined individually by each patient’s self-reported time of going to bed and getting up, were assessed separately. To consider ABP recordings valid, at least 70% successful readings during daytime and nocturnal periods were required [4]. Further information can be found in Appendix Table 2. Hypertension was defined as 24-h BP ≥ 130/80 mmHg and/or on antihypertensive drug treatment.

The present analysis focused on ambulatory SBP since, compared to DBP, is a better predictor of CVD events in older patients [6, 7]. Also, HR and nocturnal-SBP were evaluated, as both are associated with nonfatal or fatal CVD events [9, 10]. We used the relative percentage of SBP fall during the night [(daytime SBP—nighttime SBP)/daytime SBP] * 100 as estimate of nocturnal BP dipping [37].

### Assessment of covariates

At the beginning of the study, participants reported their sex, age, educational level (≤ primary, secondary, and university education) and smoking status (never, former, and current). Body mass index (BMI) was calculated as measured weight (kg) divided by squared height (m) and categorized as normal (< 25 kg/m^2^), overweight (25–29.9 kg/m^2^) and obese (≥ 30 kg/m^2^). Total energy intake (kcal/day) was calculated using standard food composition tables [33]. CVD was ascertained by asking patients for any previous physician-based diagnosis of acute myocardial infarction, stroke, or heart failure; diabetes mellitus was defined as any previous diagnoses of diabetes; and dyslipidaemia as non-fasting total cholesterol > 200 mg/dL or current use of lipid-lowering medication. Lastly, the number of antihypertensive drugs used was also collected and verified against drug packages during the home visit.

### Statistical analysis

Linear regression models were used to estimate mean differences (95% confidence interval) in daytime, nighttime, and 24-h SBP (mmHg), HR (bpm) and nocturnal-SBP fall (or dipping, %) across quintiles of MEDLIFE score; the lowest quintile was used as reference. Two sequential models were fitted. Model 1: including sex, age, and level of education; and model 2: additionally adjusting for: smoking status (never, former, and current), BMI (normal, overweight, and obese), total energy intake (continuous), prevalent CVD, diabetes, hypercholesterolemia (all dichotomous), and number of antihypertensive drugs (continuous). Linear trend tests were performed by using the quintiles of MEDLIFE score as a continuous variable. Analyses were also conducted for each 2-point increment in MEDLIFE score.

To characterize the dose–response between MEDLIFE and SBP and HR, we used restricted cubic spline analysis with 3 knots, adjusting for all covariates in model 2. To assess the independent association of each block of the MEDLIFE index, we replicated the main analyses for 1-point increment in each block, using model 2 and additionally adjusting for the remaining blocks. Similar analyses were performed for each MEDLFE item, adjusting for the remaining items. In addition, we calculated the false discovery rate of 5% for multiple comparisons using the Benjamini-Hoschberg procedure [38].

In sensitivity analyses, the main results were stratified by categories of all covariates in model 2, as well as by hypertension status (hypertensive/non-hypertensive) and antihypertensive-drug treatment status (treated/untreated), and we tested if the results varied across strata using interaction product-terms. Finally, based on previous research [39, 40], we evaluated two additional models. Model 3 was adjusted for all covariates in model 2 plus 24-h SBP, for the analysis of the association between MEDLIFE index and daytime-, nighttime-, 24-h-HR, and nocturnal-SBP dipping. Model 4 was additionally adjusted to model 2 for daytime SBP for the association between the MEDLIFE index and nighttime SBP.

Analyses were conducted with Stata version 15.0 (StataCorp LLC, College Station, Texas). P-values were two-sided and considered statistically significant at *p* < 0.05.

## Results

### Study population characteristics

From the initial 3,273 participants,we excluded: 675 due to missing data on 24-h SBP or HR; 206 for not having ≥ 70% of valid ABPM readings; and 208 because of lacking data on covariates. Thus, the analytical sample comprised 2,184 individuals (Figure s1).

Study participants had a mean age of 71.4 (± 4.3) years, 51% were women, 51.5% had never smoked, and 62.6% had primary education or less. Also 1,563 (71.57%) patients had ambulatory hypertension. Among study participants, mean daytime, nighttime, and 24-h SBP were 129.3 (± 12.6), 119.6 (± 14.6), and 126.6 (± 12.3) mmHg, respectively. The corresponding values for HR were 70.4 (± 9.4), 61.1 (± 8.4) and 67.7 (± 8.7) bpm. Mean nocturnal-SBP fall was 7.4 (± 7.9) %. Mean MEDLIFE score was 14.6 (± 2.6) points and ranged from 6 to 25. Participants with a higher adherence to MEDLIFE tended to be younger, were less frequently current smokers, and used fewer antihypertensive drugs compared to participants with a lower MEDLIFE adherence (Table 1).

**Table 1:** Characteristics of participants in the Seniors ENRICA-2 cohort according to quintiles of the MEDLIFE index

			MEDLIFE Adherence				
	Total	Q1	Q2	Q3	Q4	Q5	*p*-value
*n* (percentage)	2,184	472 (21.61)	606 (27.75)	319 (14.61)	506 (23.17)	281 (12.87)	
Sex, men	1,063(48.67)	225 (47.67)	291 (48.02)	162 (50.78)	252 (49.80)	133 (47.33)	0.857
Age, years	71.42 ± 4.31	72.07 ± 4.72	71.71 ± 4.30	71.46 ± 4.24	70.81 ± 4.08	70.75 ± 3.86	**0.002**
Educational level							0.128
≤ Primary	1,368 (62.64)	303 (64.19)	395 (65.18)	191 (59.87)	298 (58.89)	181 (64.41)	
Secondary	410 (18.77)	98 (20.76)	102 (16.83)	66 (20.69)	99 (19.57)	45 (16.01)	
University	406 (18.59)	71 (15.04)	109 (17.99)	62 (19.44)	109 (21.54)	55 (19.57)	
Smoking status							**0.006**
Current	199 (9.11)	56 (11.86)	67 (11.06)	23 (7.21)	36 (7.11)	17 (6.05)	
Former	861 (39.42)	167 (35.38)	218 (35.97)	138 (43.26)	221 (43.68)	117 (41.64)	
Never	1,124 (51.47)	249 (52.75)	321 (52.97)	158 (49.53)	249 (49.21)	147 (52.31)	
BMI, kg/m^2^							0.467
< 25	595 (27.24)	117 (24.79)	164 (27.06)	94 (29.47)	138 (27.27)	82 (29.18)	
> 25 – 29.9	1,038 (47.53)	227 (48.09)	275 (45.38)	156 (48.90)	243 (48.02)	137 (48.75)	
≥ 30	551 (25.23)	128 (27.12)	167 (27.56)	69 (21.63)	125 (24.70)	62 (22.06)	
LDL cholesterol, mg/dL	113.69 (28.96)	115.44 (28.72)	112.54 (29.28)	112.42 (29.07)	113.49 (29.69)	115.04 (27.14)	0.538
HDL cholesterol, mg/dL	53.89 (14.23)	54.15 (14.53)	52.78 (13.47)	53.03 (14.79)	54.59 (14.01)	55.58 (14.91)	0.173
Waist-to-hip ratio^a^	0.93 (0.09)	0.94 (0.09)	0.94 (0.09)	0.93 (0.09)	0.93 (0.09)	0.92 (0.09)	0.857
Total energy intake	1,955 ± 352	1,917 ± 358	1,943 ± 352	1,941 ± 355	1,995 ± 348	1,987.7 ± 340	0.890
Prevalent diseases							
CVD ^b^	71 (3.25)	21 (4.45)	16 (2.64)	8 (2.51)	18 (3.56)	8 (2.85)	0.444
Myocardial infarction	23 (32.39)	5 (21.74)	3 (13.04)	2 (8.70)	6 (26.09)	7 (30.43)	0.091
Stroke	20 (28.17)	5 (25.00)	7 (35.00)	3 (15.00)	5 (25.00)	0 (0.00)	0.538
Heart failure	36 (50.70)	13 (36.11)	10 (27.78)	4 (11.11)	7 (19.44)	2 (5.56)	0.226
Diabetes ^c^	395 (18.09)	85 (18.01)	128 (21.12)	60 (18.81)	81 (16.01)	41 (14.59)	0.104
Duration of diabetes (years)	9.82 (12.80)	12.41 (19.26)	9.55 (11.54)	9.10 (8.56)	8.73 (9.36)	8.4 + (10.45)	**<0.001**
Dyslipidaemia ^d^	1,542 (70.60)	341 (72.25)	413 (68.15)	221 (69.28)	359 (70.95)	208 (74.02)	0.373
Nº antihypertensive drugs	0.84 ± 0.95	0.83 ± 0.95	0.90 ± 1.00	0.88 ± 0.92	0.81 ± 0.93	0.71 ± 0.86	**0.033**
Use of statins	932 (42.67)	197 (21.14)	255 (27.36)	145 (15.56)	212 (22.75)	123 (13.20)	0.822
Use of antidiabetic drugs^e^	328 (15.02)	73 (22.26)	106 (32.32)	54 (16.46)	61 (18.60)	34 (10.37)	0.055
Oral antidiabetic	318 (14.56)	69 (21.70)	105 (33.02)	53 (16.67)	59 (18.55)	32 (10.06)	**0.033**
Insulin	40 (1.83)	8 (20)	13 (32.5)	6 (15)	9 (22.5)	4 (10)	0.957
Ambulatory 24-h HTN ^f^	1,561 (71.47)	342 (72.46)	454 (74.92)	235 (73.67)	339 (67.00)	191 (67.97)	**0.025**
Ambulatory SBP							
Daytime SBP, mmHg	129.33 ± 12.64	130.03 ± 13.02	129.83 ± 12.70	129.07 ± 11.76	128.80 ± 13.36	128.32 ± 11.39	**0.011**
Nighttime SBP, mmHg	119.62 ± 14.57	120.93 ± 14.96	120.16 ± 14.61	120.08 ± 14.83	119.06 ± 14.74	116.75 ± 12.81	**0.049**
24-h SBP, mmHg	126.57 ± 12.34	127.33 ± 12.85	127.08 ± 12.36	126.47 ± 11.81	126.06 ± 12.91	125.18 ± 10.83	**0.009**
Ambulatory HR							
Daytime HR, bpm	70.38 ± 9.39	71.19 ± 9.79	71.02 ± 9.54	70.29 ± 9.18	69.43 ± 9.19	69.43 ± 8.81	0.293
Nighttime HR, bpm	61.11 ± 8.36	62.36 ± 9.20	61.53 ± 8.09	61.14 ± 8.30	60.06 ± 8.19	59.96 ± 7.46	**0.002**
24-h HR, bpm	67.73 ± 8.72	68.57 ± 9.20	68.26 ± 8.81	67.68 ± 8.53	66.81 ± 8.49	66.88 ± 8.09	0.139
Nocturnal SBP fall, %	7.40 ± 7.92	6.92 ± 7.71	7.35 ± 7.88	6.91 ± 8.25	7.41 ± 7.99	8.87 ± 7.72	0.702
MEDLIFE score (0–29 points)	14.55 ± 2.59	11.02 ± 1.14	13.52 ± 0.50	15 ± 0	16.44 ± 0.50	18.80 ± 1.04	** < 0.001**
Block 1: D*iet (*0–14 points)	5.78 ± 1.67	4.06 ± 1.10	5.25 ± 1.12	6.06 ± 1.08	6.69 ± 1.19	7.84 ± 1.30	** < 0.001**
Block 2: D*ietary habits* (0–8 points)	5.10 ± 1.04	4.40 ± 0.93	4.87 ± 0.91	5.17 ± 0.90	5.52 ± 0.87	5.97 ± 0.95	** < 0.001**
Block 3: L*ifestyle habits (*0–7 points)	3.67 ± 1.32	2.57 ± 1.04	3.40 ± 1.12	3.77 ± 1.03	4.23 ± 1.11	4.99 ± 1.04	** < 0.001**

Boldface indicates statistical significance (*p* < 0.05)

Abbreviations: *BMI*, body mass index; *CVD*, cardiovascular disease; *HDL*, high density lipoprotein; *HTN*, hypertension; *LDL*, low density lipoprotein; *n*, number of participants; *Q*, quintiles

^a^ Prevalent CVD: defined as any previous diagnoses of acute myocardial infarction, stroke, or heart failure

^b^ Prevalent Diabetes: defined as any previous diagnoses of diabetes

^c^ Prevalent Dyslipidaemia: defined as antihyperlipidemic drugs use recorded on medical history or as a non-fasting total cholesterol > 200 mg/dL

^d^ Antidiabetic drugs include oral antidiabetics and insulin

^e^ Ambulatory 24-h HTN: defined as 24-h systolic blood pressure ≥ 130 mmHg and/or diastolic blood pressure ≥ 80 mmHg and/or taking antihypertensive medication

Continuous variables are expressed as mean ± standard deviation; categorical variables are expressed as frequency (percentage). p-values: Continuous variables were compared across categories of MEDLIFE using ANOVA and categorical variables were compared using chi-squared tests

### MEDLIFE and 24-h blood pressure and heart rate

Participants in the highest quintile (vs. Q1) had a lower nighttime SBP (-3.17 mmHg [95% CI: -5.25, -1.08]) and a greater nocturnal-SBP fall (1.67% [0.51, 2.83]). Although the rest of the associations with SBP did not reach statistical significance, there was a tendency towards lower values of SBP when MEDLIFE adherence increased. In addition, each 2-point increment in the MEDLIFE score was associated with lower mean (95% CI) daytime, nighttime, and 24-h HR of -0.66 bpm (-0.96, -0.37), -0.67 bpm (-0.93, -0.41) and -0.62 bpm (-0.89, -0.35), respectively, and a lower nighttime SBP of -0.59 mmHg (-1.05, -0.13) (Table 2). The spline models showed a clear inverse relationship of the MEDLIFE score with nighttime and 24-h SBP and with all time-periods HR and a higher MEDLIFE score was associated with higher nocturnal-SBP fall (Figure s2).

**Table 2:** Mean differences in SBP, HR, and nocturnal SBP fall across quintiles of MEDLIFE index (*n* = 2,148)

	Q1	Q2	Q3	Q4	Q5	p for trend	per + 2 points increment
MEDLIFE index, score range	6–12	13–14	15	16–17	18–25		
Daytime							
SBP, mmHg							
Model 1	1 (Ref.)	−0.12 (−1.64, 1.40)	−0.84 (−2.64, 0.95)	−0.97 (−2.56, 0.63)	−1.42 (−3.30, 0.45)	0.065	−0.40 (−0.81, 0.01)
Model 2	1 (Ref.)	−0.24 (−1.73, 1.26)	−0.61 (−2.38, 1.16)	−0.78 (−2.35, 0.79)	−1.00 (−2.85, 0.85)	0.194	−0.28 (−0.69, 0.13)
HR, bpm							
Model 1	1 (Ref.)	−0.24 (−1.34, 0.86)	−0.95 (−2.25, 0.35)	−2.03 (−3.19, −0.88)	−2.15 (−3.50, −0.79)	< 0.001	−0.70 (−1. 00, −0.40)
Model 2	1 (Ref.)	−0.21 (−−1.29, 0.86)	−0.70 (−1.97, 0.58)	** −1.86 (−2.99, −0.73)**	** −2.04 (−3.37, −0.71)**	** < 0.001**	** −0.66 (−0.96, −0.37)**
Nighttime							
SBP, mmHg							
Model 1	1 (Ref.)	−0.71 (−2.45, 1.03)	−0.61 (−2.66, 1.45)	−1.43 (−3.25, 0.40)	−3.80 (−5.94, −1.65)	0.001	−0.77 (−1.24, −0.30)
Model 2	1 (Ref.)	−0.87 (−2.56, 0.82)	−0.29 (−2.28, 1.71)	−1.16 (−2.93, 0.62)	** −3.17 (−5.25, −1.08)**	**0.011**	** −0.59 (−1.05, −0.13)**
HR, bpm							
Model 1	1 (Ref.)	−0.83 (−1.81, 0.15)	−1.17 (−2.32, −0.02)	−2.36 (−3.38, −1.33)	−2.53 (−3.73, −1.33)	< 0.001	−0.74 (−1.00, −0.47)
Model 2	1 (Ref.)	−0.83 (−1.79, 0.13)	−0.95 (−2.08, 0.19)	** −2.19 (−3.20, −1.18)**	** −2.33 (-3.52, -1.15)**	** < 0.001**	**-0.67 (-0.93, -0.41)**
24-h							
SBP, mmHg							
Model 1	1 (Ref.)	−0.18 (−1.67, 1.30)	−0.74 (−2.49, 1.02)	−1.00 (−2.55, 0.56)	−1.88 (−3.71, −0.05)	0.025	−0.46 (−0.86, −0.05)
Model 2	1 (Ref.)	−0.33 (−1.78, 1.12)	−0.49 (−2.20, 1.22)	−0.81 (−2.33, 0.72)	−1.41 (−3.20, 0.38)	0.103	−0.33 (−0.72, 0.07)
HR, bpm							
Model 1	1 (Ref.)	−0.37 (−1.39, 0.65)	−0.93 (−2.14, 0.27)	−2.01 (−3.08, −0.95)	−2.04 (−3.30, 0.78)	< 0.001	−0.66 (−0.94, −0.38)
Model 2	1 (Ref.)	−0.36 (−1.35, 0.64)	−0.70 (−1.88, 0.48)	** −1.86 (−2.90, −0.81)**	** −1.93 (−3.16, −0.69)**	** < 0.001**	** −0.62 (−0.89, −0.35)**
^a^Nocturnal fall							
SBP, %							
Model 1	1 (Ref.)	0.43 (−0.52, 1.37)	−0.11 (−1.23, 1.01)	0.33 (−0.66, 1.32)	1.85 (0.68, 3.01)	0.022	0.29 (0.04, 0.55)
Model 2	1 (Ref.)	0.46 (−0.48, 1.40)	−0.19 (−1.30, 0.92)	0.27 (−0.72, 1.25)	**1.67 (0.51, 2.83)**	0.052	0.24 (−0.01, 0.50)

Boldface indicates statistical significance (*p* < 0.05)

Abbreviations: *bpm*, beats per minute; *HR*, heart rate; *Ref*, reference; *SBP*, systolic blood pressure

^a^Nocturnal fall: calculated as: ((daytime SBP—nighttime SBP)/daytime SBP) * 100

Model 1: adjusted for sex (dichotomous), age (continuous), and educational level (categorical)

Model 2: additionally adjusted for smoking status (categorical), body mass index (categorical), total energy consumption (continuous), prevalent cardiovascular disease (dichotomous), prevalent diabetes (dichotomous), prevalent dyslipidaemia (dichotomous), and number of antihypertensive drugs (continuous)

### Blocks and items of MEDLIFE and 24-blood pressure and heart rate

One-point increment in Block 1 (Food consumption) was associated with a lower daytime SBP, as well as all time-periods HR (*p* < 0.05). One-point increment in Block 2 (Dietary habits) showed the greatest reduction in all time-periods HR and was also associated with greater nocturnal-SBP decline, although it was not associated with daytime, nighttime, or 24-h SBP values. Block 3 (Physical activity, rest, and conviviality) was associated with lower nighttime SBP (-0.50 mmHg [95% CI: -0.96, -0.04]) and HR (-0.34 bpm [-0.60, -0.08]) and greater nocturnal-SBP fall (0.32% [0.07, 0.58]) (Appendix Table 3).

**Fig. 1:**
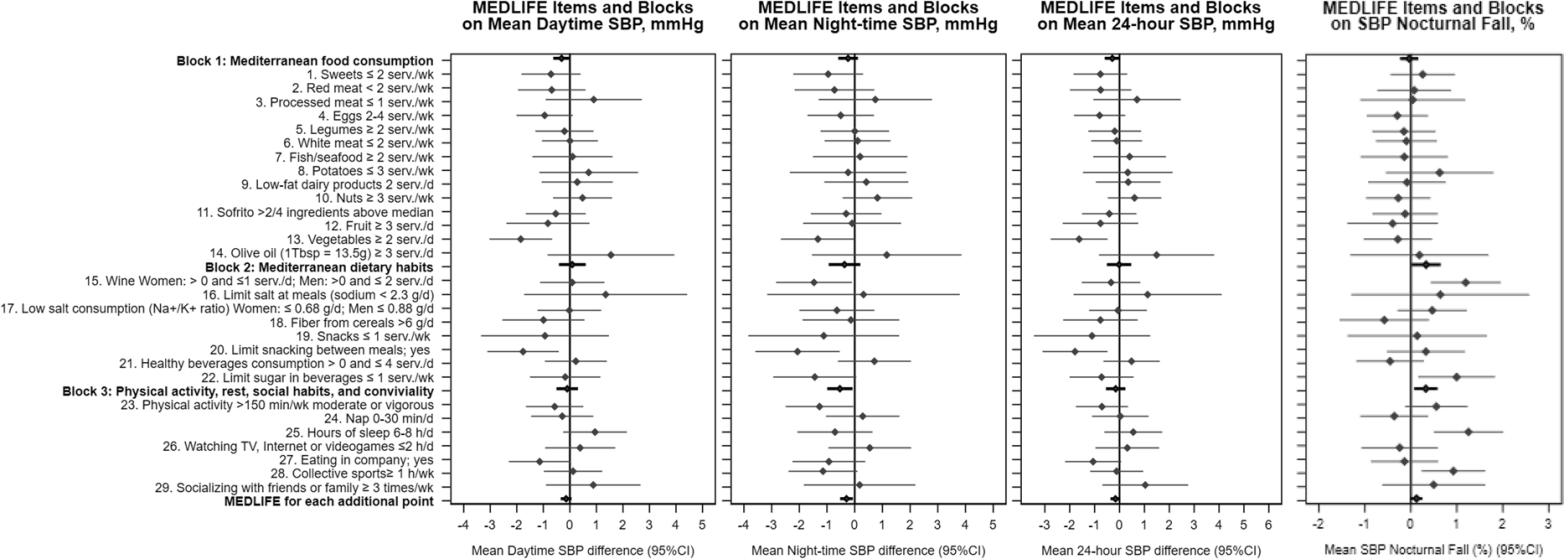
(*Title*) Mean differences in daytime, nighttime, and 24-h systolic blood pressure (mmHg) and nocturnal systolic blood pressure fall (%) and 95% CI* per 1-point increment in each MEDLIFE block and for each MEDLIFE item among Seniors ENRICA-2 participants. (*Footnote*) Abbreviations: d: day; h: hour; K + : potassium; min: minute; Na + : sodium; SBP: systolic blood pressure; serv.: servings; Tbsp: tablespoon; wk: week. Nocturnal fall: calculated as: ((daytime SBP—nighttime SBP)/daytime SBP) * 100. *Model 2: adjusted for sex (dichotomous), age (continuous), educational level (categorical), smoking status (categorical), body mass index (categorical), total energy consumption (continuous), prevalent cardiovascular disease (dichotomous), prevalent diabetes (dichotomous), prevalent dyslipidaemia (dichotomous), and number of antihypertensive drugs (continuous)

**Fig. 2:**
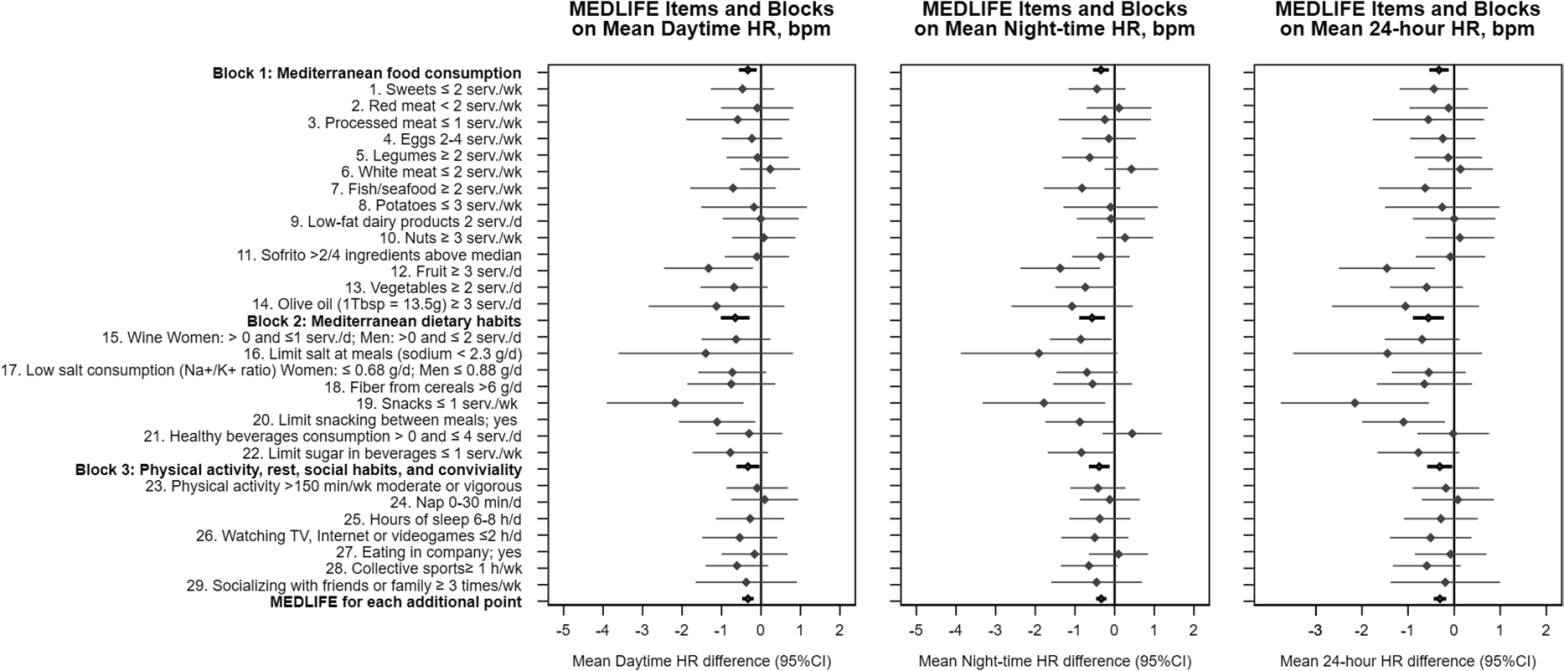
(*Title*) Mean differences in daytime, nighttime, and 24-h heart rate (bpm) and 95% CI* per 1-point increment in each MEDLIFE block and for each MEDLIFE item among Seniors ENRICA-2 participants. (*Footnote*) Abbreviations: d: day; h: hour; K + : potassium; min: minute; Na + : sodium; serv.: servings; Tbsp: tablespoon; wk: week. *Model 2: adjusted for sex (dichotomous), age (continuous), educational level (categorical), smoking status (categorical), body mass index (categorical), total energy consumption (continuous), prevalent cardiovascular disease (dichotomous), prevalent diabetes (dichotomous), prevalent dyslipidaemia (dichotomous), and number of antihypertensive drugs (continuous)

Regarding individual MEDLFE items, having at least 2 servings/day of vegetables, and limiting snacks between meals, showed lower daytime and 24-h SBP; and drinking 1 or 2 glasses/day of wine (women and men, respectively) was associated with lower nighttime SBP and a greater nocturnal-SBP fall. Also, a lower nighttime SBP was observed when physical activity recommendations were met. Hours of sleep (6–8 h) and doing physical activity in company were associated with SBP nocturnal fall (Fig. 1). Consuming ≥ 3 servings/day of fruit, limiting snacks to ≤ 1 servings/week, and limiting snacks between meals were related to lower daytime, nighttime, and 24-h HR (Fig. 2). After adjusting for multiple comparisons, the associations remained for hours of sleep and wine consumption on SBP nocturnal fall, and vegetables intake and SBP daytime (Appendix Tables 4 and 5).

### Sensitivity analyses

In analyses stratified by main covariates, no statistically significant interaction was found except one for BMI (< 25 kg/m^2^ category) in nighttime SBP (Appendix Table 6). Neither was found any effect modification by hypertension status or antihypertensive drug-treatment status (Appendix Table 7 and Appendix Table 8). Results barely changed for the association between MEDLIFE and all time-period HR, nighttime SBP or nocturnal-SBP fall after additional adjustment for 24-h SBP, and for nighttime SBP with further adjustment for daytime SBP (Appendix Table 9).

## Discussion

Among older adults living in the community, higher adherence to a Mediterranean lifestyle was associated with lower nighttime SBP, greater nocturnal-SBP fall, and lower daytime-, nighttime-, and 24-h-HR. These results have potential clinical relevance since a 2-mmHg decrease in SBP has been associated with a reduction of 6% in stroke mortality, 4% in coronary heart disease mortality, and 3% in all-cause death [41]. Likewise, a 5% attenuation in nocturnal-SBP fall has been associated with 20% increased risk of CVD death in prospective studies [42]. The magnitude of the associations we found was generally lower than expected from clinical trials but they are better at reflecting real life [4, 12, 13, 19, 20, 22, 43]. Lastly, HR has been associated with all-cause mortality in older adults, especially nighttime HR [40], and higher HR might increase the risk of coronary thrombosis, sudden death, and fatal or non-fatal myocardial infarction [44].

MEDLIFE blocks and several items of the MEDLIFE index showed independent associations with SBP and HR. The Mediterranean food consumption block was inversely associated with daytime SBP, and all time-periods HR. This concur with other studies on the effect of diet on ambulatory BP. Moore et al. [18] found that after 8-week intervention with a combination diet, participants showed lower 24-h, daytime, and nighttime ambulatory BP, independent of gender, age, ethnics, and BP-status. Also, in a recent study with 324 Chinese older adults, a 1-unit increase in DASH index was associated with 0.18 and 0.22 units lower variability in nighttime SBP and DBP, respectively [19]. Increased SBP-nocturnal fall was also found after a 4-month DASH intervention in African Americans [43]. The only investigation of the effect of MedDiet on ambulatory BP was a sub-study of the PREDIMED trial with participants at high CVD risk. The groups on MedDiet supplemented with extra-virgin olive oil or nuts showed reduced 24-h SBP and DBP compared with a control diet low in fat [20]. Plausible mechanisms of our findings include the antioxidant and anti-inflammatory properties of most components of MedDiet and DASH included in the MEDLIFE index, like fruits, vegetables, olive oil, and fibre. These foods improve endothelial function through the inhibition of the free radical damage, which react with nitric oxide to produce peroxynitrite thereby diminishing its vasodilatory effects [45]. Specifically, we found that adequate consumption of fruit was associated with lower HR. Fruit consumption has been extensively associated with reduced risk of CVD, although there are fewer studies with intermediate endpoints like heart rate, and the exact mechanism of action has not been established [46, 47].

The dietary habits block was also inversely associated with all time-periods HR and with greater nocturnal-SBP fall. Moderate wine consumption was associated with lower nighttime SBP and HR and increased nocturnal-SBP fall. These results concur with those by Jaubert et al., where very light alcohol consumption (1 drink/month to 1 drink/week) was associated with lower nighttime SBP [48]. Flavonols, resveratrol and phenolic acids present in red wine have anti-inflammatory, anti-platelet, and anti-oxidative effect and, thus, reduce BP [49]. However, our results should be taken cautiously since certain drinking patterns have detrimental effects on BP, and cross-sectional analyses cannot rule out reverse causation [50]. Additionally, low snack intake and limiting snacks between meals, were associated with lower HR. Starchy snacks have been linked to increased all-cause and CVD mortality [51]. The effect of snacking between meals highly depends on the snack pattern and frequency. While eating frequently without increasing total energy has been associated with improved lipid profile and blood pressure [52]; the quality of the snacks matter in this relationship. In our study snacks have been defined as intake of potato chips, popcorn, or other chips: ≤ 1 serving/week but the term “snack” has not been defined consistently among studies [51, 53].

Lastly, the physical activity and conviviality block was associated with lower nighttime SBP and HR, and with increased nocturnal-SBP fall. Overall, 1 additional point in block 3 (Physical activity, rest, and conviviality) of the MEDLIFE index score was associated with a 0.50 mmHg lower night-time SBP (p = 0.033), potentially yielding a 3 mmHg decrease for a score of 7 vs. 1 (i.e., 0.5 × 6). Consistently with our results, physical activity has been linked to lower ambulatory BP [54], and this beneficial effect might be greater if combined with weight management [55]. Potential mechanisms include the regulation of endothelial function due to increased nitric oxide bioavailability as response to repeated shear stress [56]. A previous study assessing a healthy lifestyle measured by MedDiet adherence and physical activity in 158 metabolically healthy older adults with excess weight showed an inverse correlation with arterial stiffness but not with DBP or SBP [57]. Our study is unique because it represents a traditional Mediterranean culture with a comprehensive number of items describing a specific way of living and while it does not include the arterial stiffness as outcome, it evaluated 24 h ABPM that is considered the most accurate and comprehensive way to measure BP, also 24 h ABPM is a stronger predictor of CVD and total mortality than office-based BP. [6, 7] In addition, Sanchez-Martinez et al. reported that social support (a variable related to conviviality) was associated with lower nighttime SBP and night/day ratio in the Seniors-ENRICA-1 cohort [39]. Also, sleep and physical activity influence BP, including nighttime BP, through variations in the autonomic nervous system [58].

The MEDLIFE index was designed to evaluate the Mediterranean lifestyle, tradition, and culture in a holistic way. Our study shows that none of the items individually could explain the magnitude of the overall association, as only some of the components were significantly associated with the outcomes. Our results add on the existing evidence, supporting the importance of diet and lifestyle combined to address BP in older adults. In addition, the joint effect observed in this study supports the inclusion of factors such as adequate rest, sociability or eating in company, and cultural and culinary choices, within public health or clinical strategies aimed to preserve cardiovascular health by promoting a Mediterranean lifestyle.

## Strengths and limitations

Strengths of this study include the large sample size, the use of validated 24-h BP devices that measure BP and HR in everyday life, the use of a validated dietary history [33], and MEDLIFE index [23, 24]. Among the limitations, the first is the cross-sectional design, which limits causal inference*.* It might also contribute to some unexpected findings; reverse causality may explain the lack of association between physical activity and lower blood pressure, because those with higher blood pressure may be more motivated to meet the recommended levels of physical activity; nevertheless, based on previous knowledge, it is more plausible that unhealthy behaviours cause elevated BP (usually asymptomatic) rather than the opposite [12]. Second, some residual confounding may persist despite extensive adjustment for factors related to BP and HR. Third, there might be measurement errors due to self-reports; however, this bias is most likely non-differential, shifting the estimates towards the null. Lastly, our study was conducted in community-living older adults in Spain, so results might not be generalized to institutionalized older people or other populations outside the Mediterranean basin; nonetheless, the diet/BP relationships seem universal though effect sizes might vary among countries.

## Conclusion 

Among older adults, higher adherence to MEDLIFE was associated with lower nighttime SBP, greater nocturnal-SBP fall, and lower HR in their everyday life. These results suggest a synergistic BP-related protection from the components of the Mediterranean lifestyle. Future studies should determine whether these results replicate in older adults from other Mediterranean and non-Mediterranean countries.

### Supplementary Information

Below is the link to the electronic supplementary material.

Supplementary file1 (DOCX 404 KB)

## Data Availability

The data that support the findings of this study are available from the corresponding author, [MSP], upon reasonable request.
