# Supplementary material for: Mediterranean lifestyle index and 24-h systolic blood pressure and heart rate in community-dwelling older adults
Source: GeroScience. 2023 Aug 10;46(1):1357–69. doi: 10.1007/s11357-023-00898-z (PMC10828285; doi:10.1007/s11357-023-00898-z)
Supplement: Supplementary file 1 — Supplementary file1 (DOCX 404 KB) [file 11357_2023_898_MOESM1_ESM.docx]

**Mediterranean Lifestyle index and 24-hour Systolic Blood Pressure and Heart Rate in Community-Dwelling Older Adults**

**Supplementary Material**

**TABLE OF CONTENT**

**Supplementary Figures:**

- **Figure s1:** Flowchart of participants in Seniors ENRICA-2 cohort.
- **Figure s2:** Dose-response association between MEDLIFE adherence (as a continuous variable) and daytime, nighttime and 24-hour mean values of systolic blood pressure (mmHg) and heart rate (bpm) in Seniors-ENRICA-2 cohort.

**Supplementary Tables:**

- **Table s1:** Description of the Mediterranean Lifestyle (MEDLIFE) index.
- **Table s2:** Protocol used for 24-hour ambulatory blood pressure monitoring.
- **Table s3:** Mean differences in SBP and HR per 1-point increment of each block of MEDLIFE.
- **Table s4.** Calculation of the 5% false discovery rate for the 29 main comparisons and daytime, nighttime, 24-hour systolic blood pressure (SBP) and SBP nocturnal fall, using the Benjamini-Hoschberg procedure.
- **Table s5.** Calculation of the 5% false discovery rate for the 29 main comparisons and daytime, nighttime, 24-hour heart rate (HR), using the Benjamini-Hoschberg procedure.
- **Table s6:** Mean differences in SBP and 24-hour-HR per 1-point increment in MEDLIFE by categories of covariates*.
- **Table s7:** Mean differences in SBP and HR according to quintiles of MEDLIFE index by hypertensive status* (n=2,148).
- **Table s8:** Mean differences in SBP and HR in participants with hypertension* by antihypertensive-drug treatment status (n=1,563).
- **Table s9:** Mean differences in HR^a^, SBP^b^ and ^c^nocturnal SBP fall between quintiles of MEDLIFE index (n=2,148).

# Appendices: Supplementary Figures

**
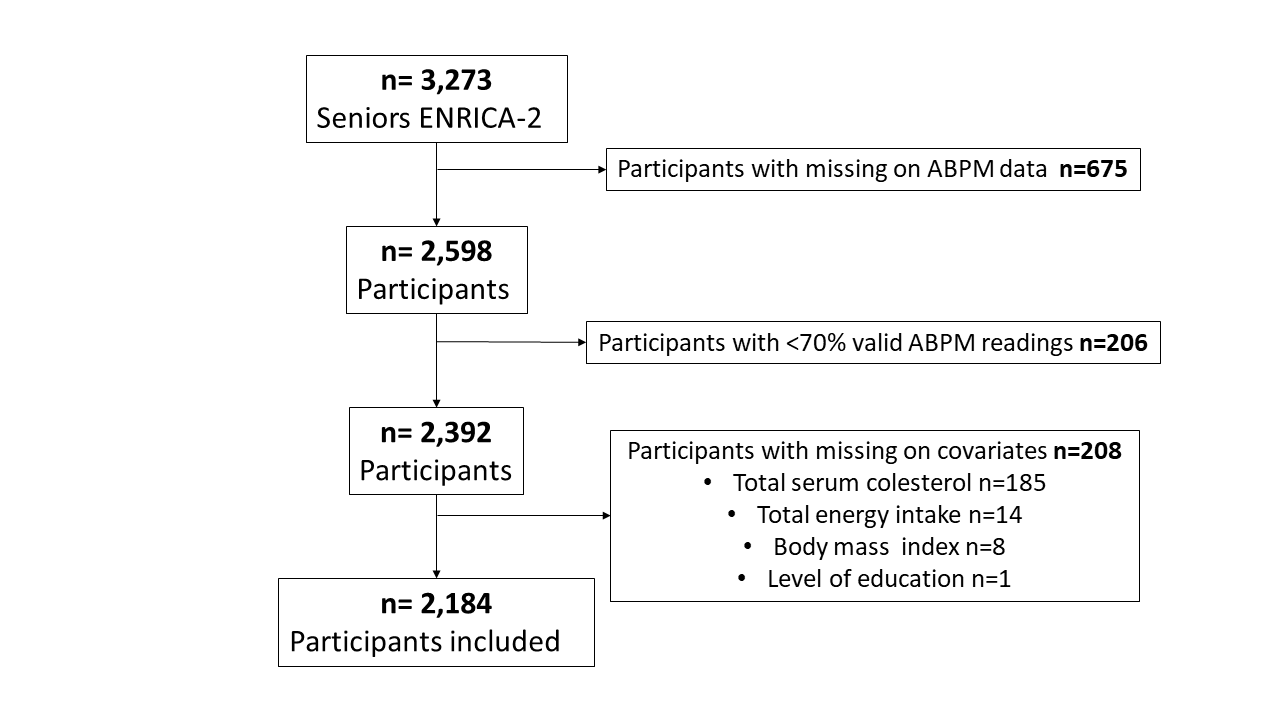
**

**Figure s1**

| **Daytime**, Systolic Blood Pressure (mmHg) | **Daytime**, Heart Rate (bpm) |
| --- | --- |
| 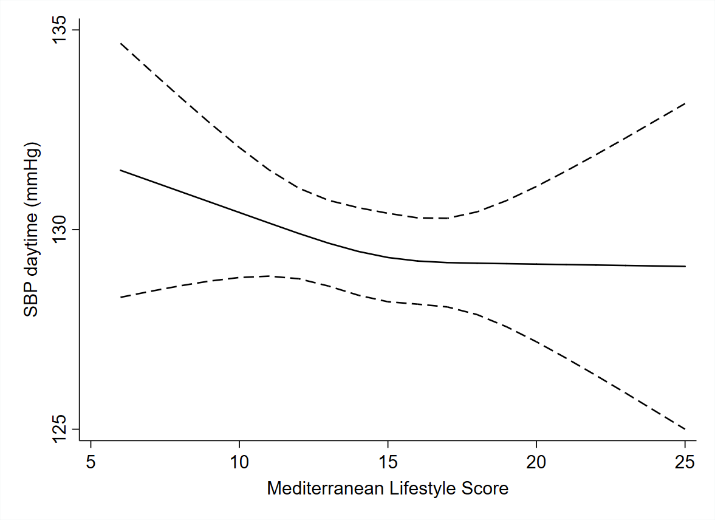 | 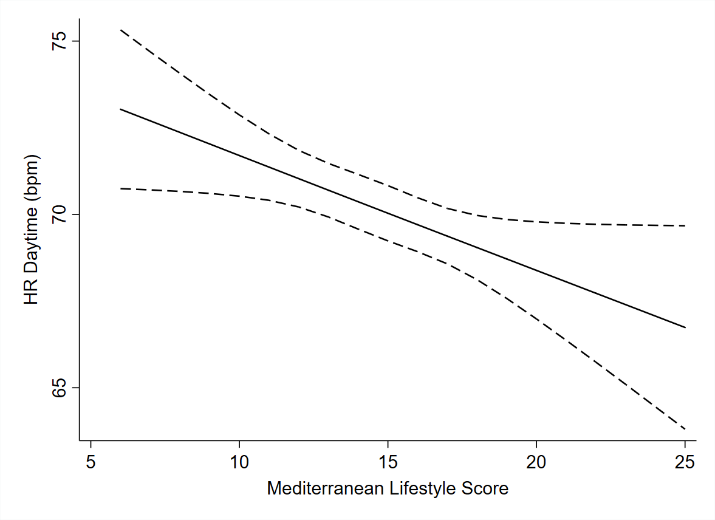 |
| **Nighttime**, Systolic Blood Pressure (mmHg) | **Nighttime**, Heart Rate (bpm) |
| 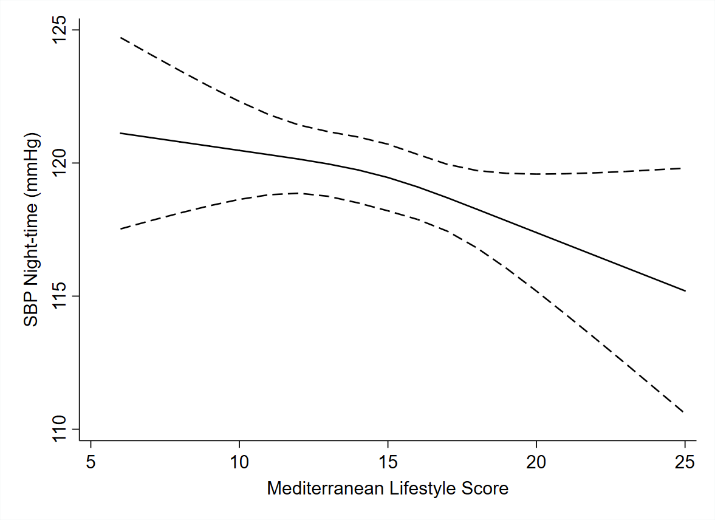 | 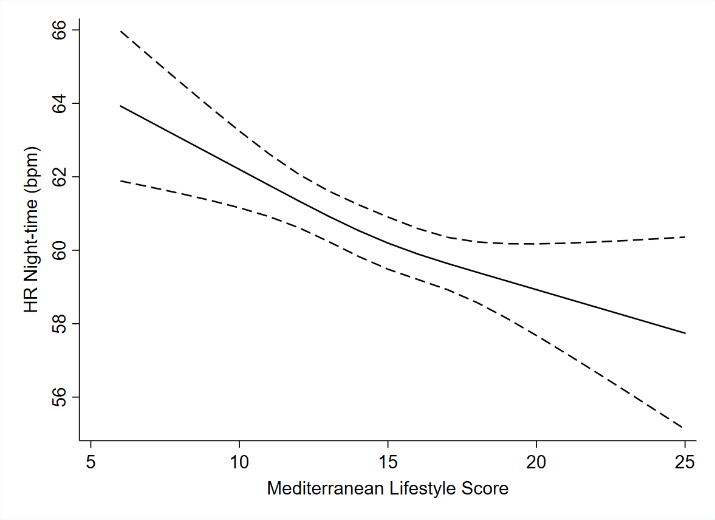 |
| **24-hour**, Systolic Blood Pressure (mmHg) | **24-hour**, Heart Rate (bpm) |
| 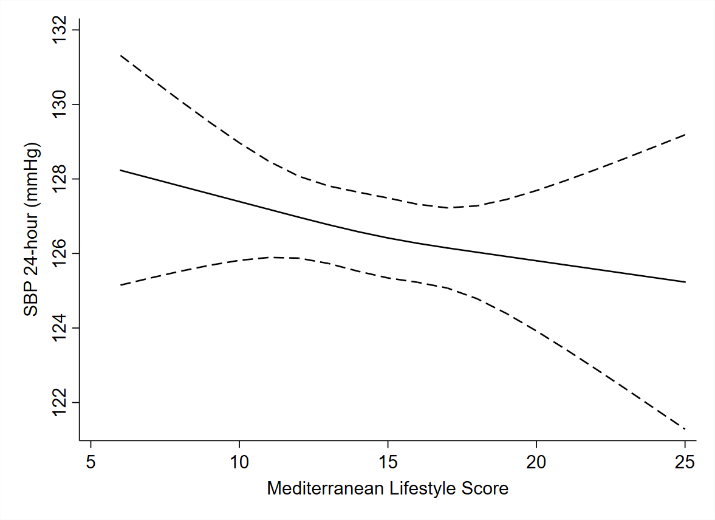 | 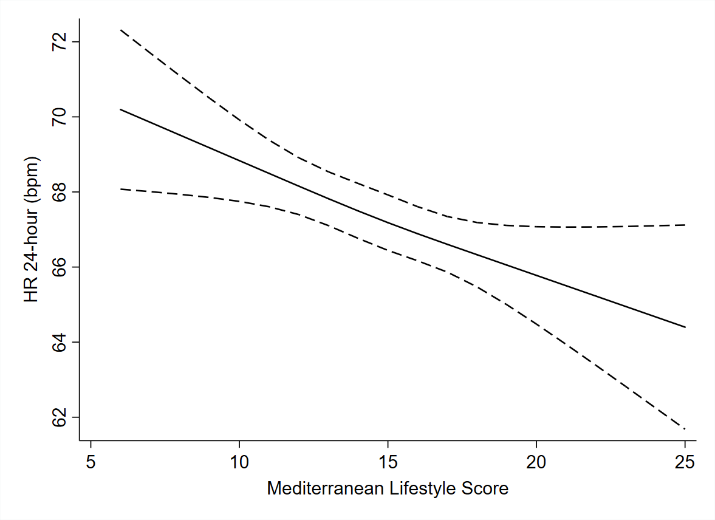 |
| **Nocturnal fall**, Systolic Blood Pressure (%) |  |
| 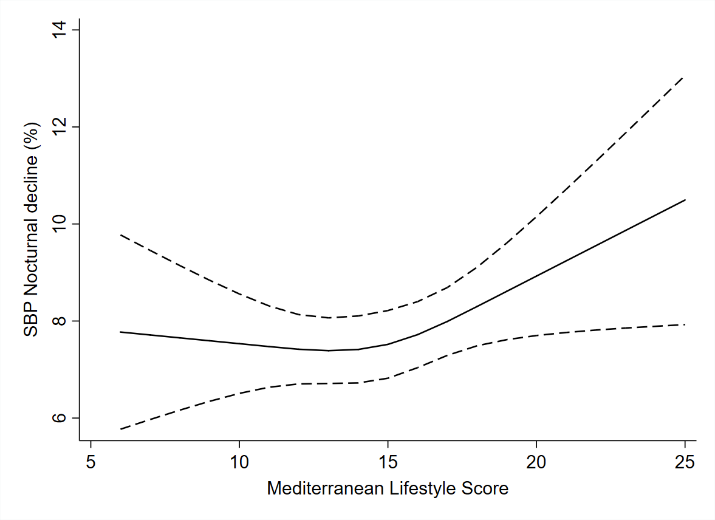 |  |

**Figure s2**

# Appendices: Supplementary Tables

**Table s1:** Description of the Mediterranean Lifestyle (MEDLIFE) index.

| **Index items** | **Components (serving size)** | **Criteria for 1 point** |
| --- | --- | --- |
| ***Block 1: Mediterranean food consumption*** | | |
| **1. Sweets** | Cookies, chocolate cookies, pastries, donuts, homemade baked goods, store-bought baked goods, muffins (50 g), chocolate (30 g), “churros” (100 g), “turrón” and marzipan (35 g) | ≤ 2 serv/wk |
| **2. Red meat** | Beef, pork, lamb (100-150 g) | < 2 serv/wk |
| **3. Processed meat** | Sausage, soft spicy sausage, bacon (50 g), cured ham (60 g), cooked ham (30 g), hamburger (150 g), liver, organ meats (100-150 g), pâté (25 g) | ≤ 1 serv/wk |
| **4. Eggs** | Eggs (1 unit = 65 g) | ≥ 2 and ≤ 4 serv/wk |
| **5. Legumes** | Lentils, beans, chickpeas, peas (150 g cooked) | ≥ 2 serv/wk |
| **6. White meat** | Chicken/turkey with skin, chicken/turkey without skin, rabbit (100-150 g) | ≤ 2 serv/wk |
| **7. Fish/seafood** | White fish, fatty fish, codfish, salted or smoked fish, shrimp, octopus, calamari (100-150 g), oysters and shellfish (6 units) | ≥ 2 serv/wk |
| **8. Potatoes** | Baked or boiled potatoes (150 g) | ≤ 3 serv/wk |
| **9. Low-fat dairy products** | Skim milk, low-fat milk (200 ml), low fat yogurt (125 g), fresh soft cheese (50 g) | 2 serv/d |
| **10. Nuts** | Almonds, peanuts, hazelnuts, walnuts (30 g) | ≥ 3 serv/w |
| **11. Sofrito** | Sauce of olive oil with onion, pepper, other vegetables (250 g) and tomato (150 g) | > 2/4 ingredients above the median |
| **12. Fruit** | Orange, banana, apple, pear, kiwi, mango, avocado, peach, apricot, nectarine (1 unit), clementine (2 units), strawberry (6 units), cherries, plums, figs, grapes (1 dessert plate), watermelon, melon (200-250 g), dates and dried fruits (150 g) | ≥ 3 serv/d |
| **13. Vegetables** | Spinach, cauliflower, broccoli, lettuce, carrot, squash, green beans, eggplant, zucchini, cucumber, pepper, asparagus, gazpacho, garden salad, other vegetables (250 g), tomato (150 g) (excludes potatoes) | ≥ 2 serv/d |
| **14. Olive oil** | Olive oil (1 Tbsp = 13.5 g) | ≥ 3 serv/d |
| ***Block 2: Dietary habits*** | | |
| **15. Wine** | Red/white wine (1 glass 100 ml) | Women: > 0 and ≤ 1 serv/d  Men: > 0 and ≤ 2 serv/d |
| **16. Limit salt at meals** | Do you add salt to foods (eggs, potatoes, fish, fried food)? Do you add salt to vegetables? Do you add salt to salads? | No |
| **17. Low salt consumption*** | Low sodium-to-potassium ratio or low sodium consumption. | Na:K Women: ≤ 0.68  Na:K Men: ≤ 0.88  Na < 2,000 mg/d |
| **18. Preference for whole grain products** | Fiber from whole grain cereals | > 6 g/d fiber from cereals |
| **19. Snacks** | Potato chips, popcorn, or other chips (50 g) | ≤ 1 serv/wk |
| **20. Limit snacking between meals** | Do you tend to snack in between meals or before going to bed? | No |
| **21. Healthy beverages consumption** | Coffee, decaffeinated coffee or tea (1 cup = 50 ml) | > 0 and ≤ 4 serv/d |
| **22. Limited consumption of sugar-sweetened beverages** | Sugar-sweetened beverages and juice (200 ml) | < 1/wk |
| ***Block 3: Physical activity, rest social habits and conviviality*** | | |
| **23. Physical activity** | Brisk walking, jogging, running, climbing stairs, bicycling, stationary cycling, swimming, dance, aerobic exercise, martial arts, gymnastics, gardening, tennis, soccer, skiing, ice skating, team sports, and other physical activities or sports | ≥ 150 min/wk moderate or  ≥ 60 min/wk vigorous |
| **24. Nap** | Napping throughout the week | 1-30 min/d |
| **25. Hours of sleep** | Sleeping throughout the week | 6-8 h/d |
| **26. Watching TV, internet, videogames** | Watching TV, using the internet or playing video games throughout the week | ≤ 2 h/d |
| **27. Eating in company** | Do you have lunch or dinner with friends, family, or others? | Yes |
| **28. Collective and non-collective sports*** | Playing soccer, tennis, squash, basketball or other team sports, running, jogging; etc. | ≥ 1 h/wk |
| **29. Socializing with friends or family** | Frequency you see family (different form who you live) or friends (home, church, bars) | ≥ 3 times/week |

***Abbreviations:*** serv: serving; d: day; h: hour; min: minute; wk: week; Tbsp: tablespoons; TV: television.

**Table s2:** Protocol used for 24-hour ambulatory blood pressure monitoring.

| Ambulatory BP readings were obtained under standardized conditions, using a validated, automated brachial oscillometric device (Mobil-OGraph 24 h PWA monitor, I.E.M., Stolberg, Germany; Mediscan/España) [1-3]. Also instructions were given to the study participants abouts the proper use of the device.  The device has already been used in previous studies during a normal day of the subject's life [4,5], and is currently used by an international consortium (i24abc.org) to which belong some of the authors of this manuscript [5].  The device was fitted in the non-dominant arm of participants, with an appropriate cuff for the arm perimeter. The device was programmed to register BP at 20-minute intervals during the day and at 30-minute intervals during the night. The time of getting-up and going-to-bed were recorded and used for analyses according to international guidelines [6]. ABPM was performed on working days with participants instructed to maintain their usual activities and to keep the arm extended and immobile at the time of each cuff inflation. As to quality control, 24-h ABPM was considered satisfactory (valid) when there were >70% valid BP readings (out of 64 possible) [2,6].  The Mobil-OGraph device has been validated in adults for measuring 24-hour heart rate, for brachial BP measurement according to recommendations of the British Hypertension Society and the European Society of Hypertension, for 24-hour brachial BP monitoring, and has received clearance from the US Food and Drug Administration and Conformité Européenne approval [2,6,7-9]. Also, regarding ambulatory measurements with the device, the reproducibility and the feasibility have been confirmed [10,11]. |
| --- |
| **References:**  [1] Franssen PML, Imholz BPM. Evaluation of the Mobil-OGraph new generation ABPM device using the ESH criteria. Blood Press Monit 2010;15:229-33.  [2] Parati G, Stergiou G, O’Brien E et al. European Society of Hypertension practice guidelines for ambulatory blood pressure monitoring. J Hypertens 2014;32:1359-66.  [3] Weber T, et al. Validation of brachial cuff for central SBP. Hypertension 2011;58:825-32.  [4] Sánchez-Martínez M, López-García E, Guallar-Castillón P, Ortolá R, García-Esquinas E, Cruz J, et al. Home and ambulatory blood pressure levels below target range and clinical effort to detect this condition: a population-based study in older treated hypertensives. Age Ageing. 2022;51(2):afab236. doi: 10.1093/ageing/afab236.  [5] Weber T, Protogerou A, Agharazii M, Argyris A, Bahous SA, Banegas JR, et al.; International Academic 24-Hour Ambulatory Aortic Blood Pressure Consortium (i24abc.org). 24-Hour Central (Aortic) Systolic BP: Reference Values and Dipping Patterns in Untreated subjects. Hypertension. 2022;79:251-260.  [6] Stergiou GS, Palatini P, Parati G et al. 2021 European Society of Hypertension practice guidelines for office and out-of-office blood pressure measurement. J Hypertens 2021;39:1293 -302.  [7] Lauder L, Scholz SS, Ewen S, Lettner C, Ukena C, Bohm M and Mahfoud F. Accuracy of pulse rate derived from 24-h ambulatory blood pressure monitoring compared with heart rate from 24-h Holter-ECG. J Hypertens. 2020;38:2387-2392.  [8] Jones CR, Taylor K, Chowienczyk P, Poston L and Shennan AH. A validation of the Mobil O Graph (version 12) ambulatory blood pressure monitor. Blood Pressure Monitoring. 2000;5:233-8.  [9] Franssen PM and Imholz BP. Evaluation of the Mobil-O-Graph new generation ABPM device using the ESH criteria. Blood pressure monitoring. 2010;15:229-31.  [10] Topouchian J, Mourad JJ, De Champvallins M, Feldmann L, Asmar R and study coordinators investigators. Feasibility of 24-h central blood pressure monitoring: experience from multinational clinical trial assessing the efficacy of perindopril/indapamide/amlodipine. J Hypertens. 2019;37:2442-2451.  [11] Protogerou AD, Argyris A, Nasothimiou E, Vrachatis D, Papaioannou TG, Tzamouranis D, Blacher J, Safar ME, Sfikakis P and Stergiou GS. Feasibility and reproducibility of noninvasive 24-h ambulatory aortic blood pressure monitoring with a brachial cuff-based oscillometric device. Am. J of Hypertens. 2012;25:876-82. |

**Table s3:** Mean differences in SBP and HR per 1-point increment of each block of MEDLIFE.

|  | ***Block 1: Diet*** | ***p-value*** | ***Block 2: Habits*** | ***p value*** | ***Block 3: Lifestyle*** | ***p value*** |
| --- | --- | --- | --- | --- | --- | --- |
| ***Daytime*** | | | | | | |
| **SBP,** mmHg |  |  |  |  |  |  |
| Model 2 | **-0.32 (-0.64, -5x10^-3^)** | **0.047** | 0.18 (-0.33, 0.69) | 0.488 | -0.07 (-0.48, 0.34) | 0.733 |
| **HR,** bpm |  |  |  |  |  |  |
| Model 2 | **-0.26 (-0.49, -0.03)** | **0.028** | **-0.57 (-0.94, -0.20)** | **0.002** | -0.28 (-0.57, 0.01) | 0.062 |
| ***Nighttime*** | | | | | | |
| **SBP,** mmHg |  |  |  |  |  |  |
| Model 2 | -0.18 (-0.54, 0.19) | 0.341 | -0.29 (-0.87, 0.29) | 0.324 | **-0.50 (-0.96, -0.04)** | **0.033** |
| **HR,** bpm |  |  |  |  |  |  |
| Model 2 | **-0.28 (-0.48, -0.07)** | **0.009** | **-0.48 (-0.80, -0.15)** | **0.004** | **-0.34 (-0.60, -0.08)** | **0.011** |
| ***24-hour*** | | | | | | |
| **SBP,** mmHg |  |  |  |  |  |  |
| Model 2 | -0.29 (-0.60, 0.02) | 0.069 | 0.06 (-0.43, 0.56) | 0.799 | -0.12 (-0.52, 0.27) | 0.538 |
| **HR,** bpm |  |  |  |  |  |  |
| Model 2 | **-0.26 (-0.48, -0.05)** | **0.015** | **-0.47 (-0.82, -0.13)** | **0.006** | -0.26 (-0.53, 0.01) | 0.058 |
| ***^a^Nocturnal fall*** | | | | | | |
| **SBP,** % |  |  |  |  |  |  |
| Model 2 | -0.09 (-0.29, 0.11) | 0.374 | **0.33 (0.01, 0.65)** | **0.042** | **0.32 (0.07, 0.58)** | **0.013** |

^a^Nocturnal fall: calculated as: ((daytime SBP - nighttime SBP)/daytime SBP) * 100.

Each block consists of: Block 1 (diet): 14 items related to Mediterranean food consumption; Block 2 (habits): 8 items related to dietary habits; Block 3 (lifestyle): 7 items related to physical activity, rest, social habits, and conviviality.

Model 2: adjusted for sex (dichotomous), age (continuous), educational level (categorical), smoking status (categorical), body mass index (categorical), total energy consumption (continuous), prevalent cardiovascular disease (dichotomous), prevalent diabetes (dichotomous), prevalent dyslipidaemia (dichotomous), and number of antihypertensive drugs (continuous).

Boldface indicates statistical significance (p<0.05). Abbreviations: HR: heart rate; SBP: systolic blood pressure.

**Table s4.** Calculation of the 5% false discovery rate for the 29 main comparisons and daytime, nighttime, 24-hour systolic blood pressure (SBP) and SBP nocturnal fall, using the Benjamini-Hoschberg procedure.

| **SBP DAYTIME** | | | |  | **SBP NIGHTIME** | | | |  | **SBP 24h** | | | |  | **SBP NOCTURNAL FALL** | | | |
| --- | --- | --- | --- | --- | --- | --- | --- | --- | --- | --- | --- | --- | --- | --- | --- | --- | --- | --- |
| **i** | **Comparison (items del medlife)** | **0.05i/29** | **p_i_** |  | **i** | **Comparison (items del medlife)** | **0.05i/29** | **p_i_** |  | **i** | **Comparison (items del medlife)** | **0.05i/29** | **p_i_** |  | **i** | **Comparison (items del medlife)** | **0.05i/29** | **p_i_** |
| 1 | Vegetables | **0,002** | **0,002** |  | 1 | Limit snacking between meals | 0,002 | 0,008 |  | 1 | Limit snacking between meals | 0,002 | 0,008 |  | 1 | Hours of sleep | **0,002** | **0,001** |
| 2 | Limit snacking between meals | 0,003 | 0,010 |  | 2 | Wine | 0,003 | 0,035 |  | 2 | Wine | 0,003 | 0,035 |  | 2 | Wine | **0,003** | **0,002** |
| 3 | Eating in company | 0,005 | 0,055 |  | 3 | Physicial activity | 0,005 | 0,042 |  | 3 | Physicial activity | 0,005 | 0,042 |  | 3 | Collective and sports | 0,005 | 0,009 |
| 4 | Eggs | 0,007 | 0,079 |  | 4 | Vegetables | 0,007 | 0,051 |  | 4 | Vegetables | 0,007 | 0,051 |  | 4 | Limited consumption of sugar-sweetened beverages | 0,007 | 0,019 |
| 5 | Hours of sleep | 0,009 | 0,122 |  | 5 | Limited consumption of sugar-sweetened beverages | 0,009 | 0,060 |  | 5 | Limited consumption of sugar-sweetened beverages | 0,009 | 0,060 |  | 5 | Physicial activity | 0,009 | 0,11 |
| 6 | Olive oil | 0,010 | 0,204 |  | 6 | Collective and sports | 0,010 | 0,071 |  | 6 | Collective and sports | 0,010 | 0,071 |  | 6 | Low salt consumption | 0,010 | 0,228 |
| 7 | Preference for whole grain products | 0,012 | 0,208 |  | 7 | Sweets | 0,012 | 0,135 |  | 7 | Sweets | 0,012 | 0,135 |  | 7 | Healthy beverages | 0,012 | 0,228 |
| 8 | Sweets | 0,014 | 0,211 |  | 8 | Eating in company | 0,014 | 0,164 |  | 8 | Eating in company | 0,014 | 0,164 |  | 8 | Preference for whole grain products | 0,014 | 0,245 |
| 9 | Red meat | 0,016 | 0,293 |  | 9 | Nuts | 0,016 | 0,201 |  | 9 | Nuts | 0,016 | 0,201 |  | 9 | Potatoes | 0,016 | 0,294 |
| 10 | Physicial activity | 0,017 | 0,299 |  | 10 | Healthy beverages | 0,017 | 0,291 |  | 10 | Healthy beverages | 0,017 | 0,291 |  | 10 | Nap | 0,017 | 0,338 |
| 11 | Fruit | 0,019 | 0,300 |  | 11 | Hours of sleep | 0,019 | 0,304 |  | 11 | Hours of sleep | 0,019 | 0,304 |  | 11 | Socializing with friends or family | 0,019 | 0,385 |
| 12 | Processed meat | 0,021 | 0,330 |  | 12 | Red meat | 0,021 | 0,319 |  | 12 | Red meat | 0,021 | 0,319 |  | 12 | Eggs | 0,021 | 0,388 |
| 13 | Socializing with friends or family | 0,022 | 0,331 |  | 13 | Low salt consumption | 0,022 | 0,352 |  | 13 | Low salt consumption | 0,022 | 0,352 |  | 13 | Fruit | 0,022 | 0,431 |
| 14 | Sofrito | 0,024 | 0,357 |  | 14 | Olive oil | 0,024 | 0,401 |  | 14 | Olive oil | 0,024 | 0,401 |  | 14 | Nuts | 0,024 | 0,442 |
| 15 | Limit salt at meals | 0,026 | 0,389 |  | 15 | Eggs | 0,026 | 0,409 |  | 15 | Eggs | 0,026 | 0,409 |  | 15 | Limit saniching between meals | 0,026 | 0,447 |
| 16 | Nuts | 0,028 | 0,398 |  | 16 | Snacks | 0,028 | 0,421 |  | 16 | Snacks | 0,028 | 0,421 |  | 16 | Vegetables | 0,028 | 0,457 |
| 17 | Snacks | 0,029 | 0,446 |  | 17 | Watching TV, internet, videogames | 0,029 | 0,475 |  | 17 | Watching TV, internet, videogames | 0,029 | 0,475 |  | 17 | Sweets | 0,029 | 0,472 |
| 18 | Potatoes | 0,031 | 0,455 |  | 18 | Processed meat | 0,031 | 0,476 |  | 18 | Processed meat | 0,031 | 0,476 |  | 18 | Limit salt at meals | 0,031 | 0,516 |
| 19 | Watching TV, internet, videogames | 0,033 | 0,565 |  | 19 | Low-fat dairy | 0,033 | 0,587 |  | 19 | Low-fat dairy | 0,033 | 0,587 |  | 19 | Watching TV, internet, videogames | 0,033 | 0,566 |
| 20 | Nap | 0,034 | 0,631 |  | 20 | Sofrito | 0,034 | 0,635 |  | 20 | Sofrito | 0,034 | 0,635 |  | 20 | Legumes | 0,034 | 0,667 |
| 21 | Low-fat dairy | 0,036 | 0,684 |  | 21 | Nap | 0,036 | 0,669 |  | 21 | Nap | 0,036 | 0,669 |  | 21 | Eating in company | 0,036 | 0,713 |
| 22 | Healthy beverages | 0,038 | 0,705 |  | 22 | Fish/seafood | 0,038 | 0,821 |  | 22 | Fish/seafood | 0,038 | 0,821 |  | 22 | Sofrito | 0,038 | 0,731 |
| 23 | Legumes | 0,040 | 0,715 |  | 23 | Potatoes | 0,040 | 0,825 |  | 23 | Potatoes | 0,040 | 0,825 |  | 23 | Fish/seafood | 0,040 | 0,768 |
| 24 | Limited consumption of sugar-sweetened beverages | 0,041 | 0,797 |  | 24 | Limit salt at meals | 0,041 | 0,858 |  | 24 | Limit salt at meals | 0,041 | 0,858 |  | 24 | White meat | 0,041 | 0,776 |
| 25 | Collective and sports | 0,043 | 0,831 |  | 25 | Socializing with friends or family | 0,043 | 0,862 |  | 25 | Socializing with friends or family | 0,043 | 0,862 |  | 25 | Olive oil | 0,043 | 0,808 |
| 26 | Wine | 0,045 | 0,885 |  | 26 | White meat | 0,045 | 0,864 |  | 26 | White meat | 0,045 | 0,864 |  | 26 | Low-fat dairy | 0,045 | 0,846 |
| 27 | Fish/seafood | 0,047 | 0,897 |  | 27 | Preference for whole grain products | 0,047 | 0,877 |  | 27 | Preference for whole grain products | 0,047 | 0,877 |  | 27 | Snacks | 0,047 | 0,857 |
| 28 | Low salt consumption | 0,048 | 0,976 |  | 28 | Fruit | 0,048 | 0,917 |  | 28 | Fruit | 0,048 | 0,917 |  | 28 | Red meat | 0,048 | 0,859 |
| 29 | White meat | 0,050 | 0,995 |  | 29 | Legumes | 0,050 | 0,995 |  | 29 | Legumes | 0,050 | 0,995 |  | 29 | Processed meat | 0,050 | 0,943 |

**Bold p_i_**: p_i_ ≤ 0.05i/29.

**Table s5.** Calculation of the 5% false discovery rate for the 29 main comparisons and daytime. nighttime. 24-hour heart rate (HR). using the Benjamini-Hoschberg procedure.

| **HR DAYTIME** | | | |  | **HR NIGHTIME** | | | |  | **HR 24h** | | | |
| --- | --- | --- | --- | --- | --- | --- | --- | --- | --- | --- | --- | --- | --- |
| **i** | **Comparison (items del medlife)** | **0.05i/29** | **p_i_** |  | **i** | **Comparison (items del medlife)** | **0.05i/29** | **p_i_** |  | **i** | **Comparison (items del medlife)** | **0.05i/29** | **p_i_** |
| 1 | Snacks | 0.002 | 0.014 |  | 1 | Fruit | 0.002 | 0.007 |  | 1 | Fruit | 0.002 | 0.006 |
| 2 | Fruit | 0.003 | 0.021 |  | 2 | Snacks | 0.003 | 0.024 |  | 2 | Snacks | 0.003 | 0.009 |
| 3 | Limit snacking between meals | 0.005 | 0.024 |  | 3 | Wine | 0.005 | 0.031 |  | 3 | Limit snacking between meals | 0.005 | 0.017 |
| 4 | Low salt consumption | 0.007 | 0.099 |  | 4 | Limit snacking between meals | 0.007 | 0.047 |  | 4 | Limited consumption of sugar-sweetened beverages | 0.007 | 0.086 |
| 5 | Limited consumption of sugar-sweetened beverages | 0.009 | 0.111 |  | 5 | Limited consumption of sugar-sweetened beverages | 0.009 | 0.054 |  | 5 | Wine | 0.009 | 0.092 |
| 6 | Vegetables | 0.010 | 0.116 |  | 6 | Vegetables | 0.010 | 0.057 |  | 6 | Collective and sports | 0.010 | 0.113 |
| 7 | Collective and sports | 0.012 | 0.131 |  | 7 | Limit salt at meals | 0.012 | 0.058 |  | 7 | Vegetables | 0.012 | 0.136 |
| 8 | Wine | 0.014 | 0.155 |  | 8 | Collective and sports | 0.014 | 0.072 |  | 8 | Limit salt at meals | 0.014 | 0.166 |
| 9 | Preference for whole grain products | 0.016 | 0.185 |  | 9 | Low salt consumption | 0.016 | 0.076 |  | 9 | Low salt consumption | 0.016 | 0.177 |
| 10 | Olive oil | 0.017 | 0.198 |  | 10 | Legumes | 0.017 | 0.08 |  | 10 | Olive oil | 0.017 | 0.194 |
| 11 | Fish/seafood | 0.019 | 0.2 |  | 11 | Fish/seafood | 0.019 | 0.096 |  | 11 | Fish/seafood | 0.019 | 0.219 |
| 12 | Limit salt at meals | 0.021 | 0.215 |  | 12 | Olive oil | 0.021 | 0.17 |  | 12 | Preference for whole grain products | 0.021 | 0.220 |
| 13 | Sweets | 0.022 | 0.252 |  | 13 | White meat | 0.022 | 0.219 |  | 13 | Sweets | 0.022 | 0.249 |
| 14 | Watching TV. internet. videogames | 0.024 | 0.267 |  | 14 | Sweets | 0.024 | 0.222 |  | 14 | Watching TV. internet. videogames | 0.024 | 0.255 |
| 15 | Processed meat | 0.026 | 0.376 |  | 15 | Physicial activity | 0.026 | 0.235 |  | 15 | Processed meat | 0.026 | 0.365 |
| 16 | Healthy beverages | 0.028 | 0.482 |  | 16 | Healthy beverages | 0.028 | 0.245 |  | 16 | Hours of sleep | 0.028 | 0.482 |
| 17 | Hours of sleep | 0.029 | 0.532 |  | 17 | Watching TV. internet. videogames | 0.029 | 0.247 |  | 17 | Eggs | 0.029 | 0.496 |
| 18 | White meat | 0.031 | 0.55 |  | 18 | Preference for whole grain products | 0.031 | 0.271 |  | 18 | Physicial activity | 0.031 | 0.627 |
| 19 | Eggs | 0.033 | 0.554 |  | 19 | Hours of sleep | 0.033 | 0.341 |  | 19 | Potatoes | 0.033 | 0.683 |
| 20 | Socializing with friends or family | 0.034 | 0.566 |  | 20 | Sofrito | 0.034 | 0.348 |  | 20 | White meat | 0.034 | 0.701 |
| 21 | Eating in company | 0.036 | 0.703 |  | 21 | Socializing with friends or family | 0.036 | 0.435 |  | 21 | Legumes | 0.036 | 0.730 |
| 22 | Potatoes | 0.038 | 0.795 |  | 22 | Nuts | 0.038 | 0.473 |  | 22 | Nuts | 0.038 | 0.739 |
| 23 | Sofrito | 0.040 | 0.801 |  | 23 | Processed meat | 0.040 | 0.676 |  | 23 | Socializing with friends or family | 0.040 | 0.752 |
| 24 | Physicial activity | 0.041 | 0.804 |  | 24 | Eggs | 0.041 | 0.679 |  | 24 | Red meat | 0.041 | 0.782 |
| 25 | Legumes | 0.043 | 0.829 |  | 25 | Nap | 0.043 | 0.753 |  | 25 | Sofrito | 0.043 | 0.830 |
| 26 | Nap | 0.045 | 0.829 |  | 26 | Red meat | 0.045 | 0.791 |  | 26 | Nap | 0.045 | 0.842 |
| 27 | Red meat | 0.047 | 0.841 |  | 27 | Eating in company | 0.047 | 0.797 |  | 27 | Eating in company | 0.047 | 0.844 |
| 28 | Nuts | 0.048 | 0.864 |  | 28 | Low-fat dairy | 0.048 | 0.917 |  | 28 | Fruit | 0.048 | 0.957 |
| 29 | Low-fat dairy | 0.050 | 0.986 |  | 29 | Potatoes | 0.050 | 0.995 |  | 29 | Legumes | 0.050 | 1 |

**Bold p_i_**: p_i_ ≤ 0.05i/29.

**Table s6:** Mean differences in SBP and 24-hour-HR per 1-point increment in MEDLIFE by categories of covariates*.

|  |  | **SBP Daytime** | | | **SBP Nighttime** | | | **SBP 24-hour** | | | **HR 24-hour** | | |
| --- | --- | --- | --- | --- | --- | --- | --- | --- | --- | --- | --- | --- | --- |
|  | **N (freq.)** | **MEDLIFE** | **p value** | **p for interaction** | **MEDLIFE** | **p value** | **p for interaction** | **MEDLIFE** | **p value** | **p for interaction** | **MEDLIFE** | **p value** | **p for interaction** |
| **Sex** |  |  |  | 0.903 |  |  | 0.407 |  |  | 0.757 |  |  | 0.524 |
| Men | 1,063 | -0.11 (-0.40, 0.18) | 0.467 |  | -0.19 (-0.50, 0.12) | 0.229 |  | -0.11 (-0.39, 0.16) | 0.414 |  | **-0.37 (-0.57, -0.16)** | **<0.001** |  |
| Women | 1,121 | -0.15 (-0.44, 0.14) | 0.305 |  | **-0.38 (-0.71, -0.04)** | **0.029** |  | -0.19 (-0.47, 0.09) | 0.188 |  | **-0.25 (-0.45, -0.08)** | **0.005** |  |
| **Age** |  |  |  | 0.306 |  |  | 0.964 |  |  | 0.501 |  |  | 0.783 |
| < 75 years old | 1,676 | -0.20 (-0.43, 0.03) | 0.096 |  | **-0.28 (-0.54, -0.02)** | **0.033** |  | -0.19 (-0.42, 0.03) | 0.090 |  | **-0.29 (-0.45, -0.13)** | **<0.001** |  |
| ≥ 75 years old | 508 | 3x10^-3^ (-0.44, 0.45) | 0.987 |  | -0.38 (-0.88, 0.13) | 0.143 |  | -0.09 (-0.52, 0.33) | 0.664 |  | **-0.37 (-0.64, -0.10)** | **0.007** |  |
| **Educational level** |  |  |  | 0.375 |  |  | 0.799 |  |  | 0.566 |  |  | 0.385 |
| Primary or less | 1,368 | -0.21 (-0.47, 0.04) | 0.099 |  | -0.26 (-0.55, 0.04) | 0.084 |  | -0.20 (-0.45, 0.05) | 0.111 |  | **-0.35 (-0.52, 0.18)** | **<0.001-** |  |
| Secondary | 410 | -0.12 (-0.58, 0.34) | 0.605 |  | -0.49 (-1.00, 0.02) | 0.060 |  | -0.21 (-0.65, 0.24) | 0.363 |  | 0.13 (-0.44, 0.18 | 0.408 |  |
| University | 406 | -0.19 (-0.33, 0.70) | 0.473 |  | -0.21 (-0.77, 0.35) | 0.457 |  | 0.09 (-0.40, 0.59) | 0.711 |  | **-0.36 (-0.68, -0.04)** | **0.027** |  |
| **Smoking status** |  |  |  | 0.186 |  |  | 0.097 |  |  | 0.126 |  |  | 0.970 |
| Current | 199 | -0.67 (-1.41, 0.07) | 0.077 |  | -0.85 (-1.76, 0.06) | 0.066 |  | -0.70 (-1.44, 0.05) | 0.069 |  | -0.37 (-0.85, 0.11) | 0.130 |  |
| Former | 861 | -0.01 (-0.03, 0.30) | 0.941 |  | -0.05 (-0.40, 0.29) | 0.765 |  | -0.01 (-0.31, 0.29) | 0.966 |  | **-0.30 (-0.52, -0.08)** | **0.008** |  |
| Never | 1,124 | -0.15 (-0.44, 0.13) | 0.294 |  | **-0.37 (-0.70, -0.04)** | **0.029** |  | -0.19 (-0.47, 0.09) | 0.187 |  | **-0.29 (-0.48, -0.11)** | **0.002** |  |
| **BMI** |  |  |  | 0.287 |  |  | **0.048** |  |  | 0.211 |  |  | 0.537 |
| < 25 kg/m^2^ | 595 | -0.30 (-0.68, 0.07) | 0.115 |  | **-0.71 (-1.13, -0.29)** | **0.001** |  | **-0.37 (-0.74, -0.01)** | **0.045** |  | **-0.41 (-0.65, -0.18)** | **0.001** |  |
| 25-29 kg/m^2^ | 1,038 | 0.01 (-0.28, 0.30) | 0.955 |  | -0.12 (-0.45, 0.22) | 0.496 |  | -0.02 (-0.30, 0.27) | 0.916 |  | **-0.29 (-0.49, -0.08)** | **0.006** |  |
| ≥ 30 kg/m^2^ | 551 | -0.24 (-0.67, 0.19) | 0.268 |  | -0.18 (-0.67, 0.30) | 0.459 |  | -0.21 (-0.62, 0.20) | 0.322 |  | -0.23 (-0.52, 0.05) | 0.107 |  |
| **Total energy intake** |  |  |  | 0.371 |  |  | 0.487 |  |  | 0.408 |  |  | 0.803 |
| Tertile 1 | 728 | -0.27 (-0.65, 0.11) | 0.168 |  | **-0.44 (-0.88, -0.01)** | **0.048** |  | -0.29 (-0.67, 0.08) | 0.127 |  | **-0.34 (-0.57, -0.10)** | **0.005** |  |
| Tertile 2 | 728 | -0.22 (-0.56, 0.12) | 0.205 |  | -0.31 (-0.69, 0.06) | 0.104 |  | -0.23 (-0.56, 0.10) | 0.167 |  | **-0.26 (-0.50, -0.02)** | **0.037** |  |
| Tertile 3 | 728 | 0.07 (-0.27, 0.42) | 0.675 |  | -0.14 (-0.53, 0.25) | 0.485 |  | 0.03 (-0.30, 0.36) | 0.865 |  | **-0.32 (-0.57, -0.08)** | **0.009** |  |
| **Prev. Diabetes** |  |  |  | 0.745 |  |  | 0.646 |  |  | 0.994 |  |  | 0.409 |
| No | 1,789 | -0.13 (-0.35, 0.09) | 0.250 |  | **0.33 (-0.58, -0.08)** | **0.009** |  | -0.17 (-0.38, 0.05) | 0.125 |  | **-0.33 (-0.48, -0.18)** | **<0.001** |  |
| Yes | 395 | -0.11 (-0.65, 0.42) | 0.674 |  | -0.03 (-0.66, 0.60) | 0.937 |  | -0.06 (-0.58, 0.46) | 0.814 |  | -0.22 (-0.57, 0.13) | 0.213 |  |
| **Prev. Diabetes (HbA1c)^a^** |  |  |  | 0.286 |  |  | 0.629 |  |  | 0.520 |  |  | 0.053 |
| No | 865 | 0.06 (-0.27, 0.40) | 0.722 |  | -0.11 (-0.48, 0.26) | 0.568 |  | 0.02 (-0.31, 0.34) | 0.928 |  | -**0.35 (-0.57, -0.12**) | **0.002** |  |
| Yes | 110 | -0.48 (-1.31, 0.35) | 0.252 |  | 0.08 (-0.85, 1.01) | 0.867 |  | -0.32 (-1.11, 0.46) | 0.417 |  | 0.20 (-0.46, 0.86) | 0.548 |  |
| **Prev. dyslipidaemia** |  |  |  | 0.339 |  |  | 0.703 |  |  | 0.475 |  |  | 0.114 |
| No | 642 | 0.01 (-0.38, 0.40) | 0.943 |  | -0.28 (-0.72, 0.17) | 0.220 |  | -0.06 (-0.44, 0.31) | 0.737 |  | -0.11 (-0.38, 0.16) | 0.412 |  |
| Yes | 1,542 | -0.18 (-0.42, 0.05) | 0.131 |  | **-0.30 (-0.57, -0.03)** | **0.030** |  | -0.19 (-0.42, 0.04) | 0.111 |  | **-0.38 (-0.54, -0.23)** | **<0.001** |  |
| **Prev. CVD** |  |  |  | 0.792 |  |  | 0.997 |  |  | 0.883 |  |  | 0.246 |
| No | 2,113 | -0.14 (-0.34, 0.07) | 0.190 |  | **-0.30 (-0.53, -0.06)** | **0.012** |  | -0.16 (-0.36, 0.04) | 0.112 |  | **-0.30 (-0.43, -0.16)** | **<0.001** |  |
| Yes | 71 | -0.17 (-1.56, 1.23) | 0.811 |  | -0.21 (-1.89, 1.47) | 0.799 |  | -0.14 (-1.54, 1.27) | 0.847 |  | -0.77 (-1.57, 0.02) | 0.056 |  |
| **Antihypertensive treatment** |  |  |  | 0.939 |  |  | 0.408 |  |  | 0.915 |  |  | 0.663 |
| 0 drugs | 1,003 | -0.12 (-0.40, 0.17) | 0.492 |  | **-0.36 (-0.68, -0.05)** | **0.023** |  | -0.15 (-0.43, 0.12) | 0.276 |  | **-0.25 (-0.45, -0.06)** | **0.011** |  |
| ≥ 1 drugs | 1,181 | -0.16 (-0.45, 0.13) | 0.294 |  | -0.23 (-0.57, 0.10) | 0.174 |  | -0.17 (-0.45, 0.12) | 0.247 |  | **-0.38 (-0.57, -0.19)** | **<0.001** |  |

Abbreviations: BMI: body mass index; CVD: cardiovascular disease; freq.: frequency; HR: heart rate; prev.: prevalent; SBP: systolic blood pressure. ^a^ Diabetes (HbA1c): diabetes defined as HbA1c ≥ 6.5%. *Categories of covariates included in Model 2: adjusted for sex (dichotomous), age (continuous), educational level (categorical), smoking status (categorical), body mass index (categorical), total energy consumption (continuous), prevalent cardiovascular disease (dichotomous), prevalent diabetes (dichotomous), prevalent dyslipidaemia (dichotomous), and number of antihypertensive drugs (continuous). Boldface indicates statistical significance (p<0.05).

**Table s7:** Mean differences in SBP and HR according to quintiles of MEDLIFE index by hypertensive status* (n=2,148).

| **Quintiles of MEDLIFE index score** | | | | | | | | | | | | | |
| --- | --- | --- | --- | --- | --- | --- | --- | --- | --- | --- | --- | --- | --- |
|  | **NO HYPERTENSIVE*** | | | | | | **HYPERTENSIVE*** | | | | | | |
|  | **Q1** | **Q2** | **Q3** | **Q4** | **Q5** | p for trend | **Q1** | **Q2** | **Q3** | **Q4** | **Q5** | p for trend | p for interaction |
| N (frequency) | 130 | 152 | 84 | 167 | 90 |  | 342 | 454 | 235 | 339 | 191 |  |  |
| **MEDLIFE index**, score range | 7-12 | 13-14 | 15 | 16-17 | 18-25 |  | 6-12 | 13-14 | 15 | 16-17 | 18-23 |  |  |
| ***Daytime SBP,*** *mmHg* | | | | | | | | | | | | | |
| Model 2 | 1 (Ref.) | -0.73 (-2.54, 1.08) | 0.42 (-1.69, 2.53) | -0.71 (-2.48, 1.07) | -0.12 (-02.19, 1.95) | 0.855 | 1 (Ref.) | -0.26 (-2.01, 1.48) | -1.28 (-3.35, 0.79) | -2x10^-3^ (-1.89, 1.189) | -1.46 (-3.68, 0.76) | 0.347 | 0.523 |
| ***Daytime HR****, bpm* | | | | | | | | | | | | | |
| Model 2 | 1 (Ref.) | -0.55 (-2.55, 1.45) | -0.60 (-2.93, 1.74) | -2.09 (-4.05, -0.12) | -1.28 (-3.56, 1.01) | 0.060 | 1 (Ref.) | -0.18 (-1.46, 1.09) | -0.86 (-2.38, 0.66) | **-1.78 (-3.17, -0.40)** | **-2.48 (-4.11, -0.86)** | **<0.001** | 0.907 |
| ***Nighttime SBP,*** *mmHg* | | | | | | | | | | | | | |
| Model 2 | 1 (Ref.) | -2.01 (-4.22, 0.19) | -1.56 (-4.14, 1.01) | -1.30 (-3.46, 0.87) | **-3.81 (-6.33, -1.28)** | **0.031** | 1 (Ref.) | -0.62 (-2.63, 1.39) | -0.11 (-2.50, 2.28) | -0.32 (-2.50, 1.86) | **-2.98 (-.5.54, -0.42)** | 0.109 | 0.615 |
| ***Nighttime HR****, bpm* | | | | | | | | | | | | | |
| Model 2 | 1 (Ref.) | -1.82 (-3.69, 0.05) | -1.57 (-3.75, 0.61) | -**2.49 (-4.33, -0.66)** | -1.59 (-3.72, 0.55) | 0.057 | 1 (Ref.) | -0.57 (-1.69, 0.54) | -0.89 (-2.21, 0.44) | **-2.10 (-3.31, -0.90)** | **-2.80 (-4.22, -1.38)** | **<0.001** | 0.494 |
| ***24h SBP,*** *mmHg* | | | | | | | | | | | | | |
| Model 2 | 1 (Ref.) | -1.06 (-2.72, 0.61) | -0.27 (-2.22, 1.67) | -0.62 (-2.26, 1.02) | -0.90 (-2.81, 1.00) | 0.556 | 1 (Ref.) | -0.26 (-1.95, 1.43) | -0.86 (-2.88, 1.15) | -0.09 (-1.92, 1.74) | -1.72 (-3.88, 0.43) | 0.243 | 0.611 |
| ***24h HR****, bpm* | | | | | | | | | | | | | |
| Model 2 | 1 (Ref.) | -0.84 (-2.72, 1.04) | -0.85 (-3.04, 1.34) | -1.98 (-3.83, -0.14) | -1.09 (-3.24, 1.06) | 0.095 | 1 (Ref.) | -0.28 (-1.46, 0.89) | -0.78 (-2.18, 0.62) | **-1.84 (-3.11, 0.56)** | **-2.40 (-3.90, -0.91)** | **<0.001** | 0.681 |
| ***^a^Nocturnal SBP fall****, %* | | | | | | | | | | | | | |
| Model 2 | 1 (Ref.) | 1.04 (-0.69, 2.76) | 1.53 (-0.49, 3.54) | 0.40 (-1.30, 2.10) | **2.93 (-0.95, 4.91)** | **0.049** | 1 (Ref.) | 0.24 (-0.88, 1.37) | .0.80 (-2.13, 0.54) | 0.17 (-1.04, 1.39) | 1.15 (-0.28, 2.58) | 0.303 | 0.158 |

^a^Nocturnal SBP fall: calculated as: ((daytime SBP - nighttime SBP)/daytime SBP) * 100.

Abbreviations: ABPM: ambulatory blood pressure monitoring; HR: heart rate; Ref.: reference; SBP: systolic blood pressure. *Hypertensive status: defined as 24-hour systolic blood pressure ≥130 mmHg and/or diastolic blood pressure ≥80 mmHg and/or taking antihypertensive medication. Model 2: adjusted for sex (dichotomous), age (continuous), educational level (categorical), smoking status (categorical), body mass index (categorical), total energy consumption (continuous), prevalent cardiovascular disease (dichotomous), prevalent diabetes (dichotomous), prevalent dyslipidaemia (dichotomous), and number of antihypertensive drugs (continuous).

Boldface indicates statistical significance (p<0.05).

**Table s8:** Mean differences in SBP and HR in participants with hypertension* by antihypertensive-drug treatment status (n=1,563).

| **Quintiles of MEDLIFE index score** | | | | | | | | | | | | | |
| --- | --- | --- | --- | --- | --- | --- | --- | --- | --- | --- | --- | --- | --- |
|  | **UNTREATED** | | | | | | **TREATED** | | | | | | |
|  | **Q1** | **Q2** | **Q3** | **Q4** | **Q5** | p for trend | **Q1** | **Q2** | **Q3** | **Q4** | **Q5** | p for trend | p for interaction |
| N (frequency) | 86 | 101 | 54 | 88 | 53 |  | 254 | 336 | 185 | 267 | 139 |  |  |
| **MEDLIFE index**, score range | 9-12 | 13-14 | 15 | 16-17 | 18-22 |  | 6-12 | 13-14 | 15 | 16-17 | 18-23 |  |  |
| ***Daytime SBP,*** *mmHg* | | | | | | | | | | | | | |
| Model 2 | 1 (Ref.) | 0.65 (-2.42, 3.72) | 0.80 (-2.87, 4.46) | 0.98 (-2.22, 4.18) | -1.66 (-5.35, 2.03) | 0.648 | 1 (Ref.) | -0.77 (-2.86, 1.32) | -1.75 (-4.18, 0.69) | -0.60 (-2.84, 1.63) | -1.49 (-4.15, 1.18) | 0.398 | 0.828 |
| ***Daytime HR****, bpm* | | | | | | | | | | | | | |
| Model 2 | 1 (Ref.) | 0.02 (-2.63, 2.67) | 0.19 (-2.96, 3.34) | -0.68 (-3.43, 2.07) | 1.24 (-1.94, 4.42) | 0.771 | 1 (Ref.) | -0.06 (-1.55, 1.43) | -0.96 (-2.70, 0.77) | **-2.40 (-3.99, -0.81)** | **-2.77 (-4.67, -0.88)** | **<0.001** | 0.226 |
| ***Nighttime SBP,*** *mmHg* | | | | | | | | | | | | | |
| Model 2 | 1 (Ref.) | 0.68 (-3.08, 4.44) | 0.80 (-3.69, 5.28) | -0.42 (-4.34, 3.50) | **-4.55 (-9.07, -0.02)** | 0.083 | 1 (Ref.) | -0.88 (-3.29, 1.52) | -0.94 (-3.74, 1.86) | -0.85 (-3.42, 1.72) | -2.93 (-5.99, 0.14) | 0.133 | 0.408 |
| ***Nighttime HR****, bpm* | | | | | | | | | | | | | |
| Model 2 | 1 (Ref.) | -1.16 (-3.39, 1.07) | -0.74 (-3.40, 1.92) | -1.25 (-3.57, 1.08) | -0.39 (-3.07, 2.30) | 0.651 | 1 (Ref.) | -0.50 (-1.81, 0.81) | -1.15 (-2.67, 0.37) | **-2.56 (-3.96, -1.16)** | **-2.67 (-4.33, -1.00)** | **<0.001** | 0.452 |
| ***24h SBP,*** *mmHg* | | | | | | | | | | | | | |
| Model 2 | 1 (Ref.) | 1.05 (-1.94, 4.03) | 1.06 (-2.49, 4.62) | 1.02 (-2.09, 4.13) | -1.87 (-5.46, 1.72) | 0.525 | 1 (Ref.) | -0.73 (-2.77, 1.32) | -1.48 (-3.86, 0.89) | -0.67 (-2.85, 1.51) | -1.77 (-4.37, 0.83) | 0.248 | 0.833 |
| ***24h HR****, bpm* | | | | | | | | | | | | | |
| Model 2 | 1 (Ref.) | -0.35 (-2.76, 2.06) | -0.07 (-2.94, 2.81) | -0.63 (-3.14, 1.89) | 0.92 (-1.99, 3.82) | 0.771 | 1 (Ref.) | -0.18 (-1.56, 1.19) | -0.90 (-2.50, 0.70) | **-2.42 (-3.89, -0.95)** | **-2.56 (-4.30, -0.81)** | **<0.001** | 0.210 |
| ***^a^Nocturnal SBP fall****, %* | | | | | | | | | | | | | |
| Model 2 | 1 (Ref.) | 2x10^-3^ (-2.17, 2.18) | 0.03 (-2.56, 2.62) | 1.09 (-1.17, 3.36) | 2.40 (-0.21, 5.02) | 0.055 | 1 (Ref.) | 0.08 (-1.25, 1.41) | -0.52 (-2.07, 1.03) | 0.12 (-1.30, 1.54) | 1.02 (-0.68, 2.71) | 0.406 | 0.285 |

^a^Nocturnal SBP fall: calculated as: ((daytime SBP - nighttime SBP)/daytime SBP) * 100.

Abbreviations: ABPM: ambulatory blood pressure monitoring; HR: heart rate; Ref.: reference; SBP: systolic blood pressure. *Hypertension defined as 24-hour systolic blood pressure ≥130 mmHg and/or diastolic blood pressure ≥80 mmHg and/or taking antihypertensive medication. Model 2: adjusted for sex (dichotomous), age (continuous), educational level (categorical), smoking status (categorical), body mass index (categorical), total energy consumption (continuous), prevalent cardiovascular disease (dichotomous), prevalent diabetes (dichotomous), prevalent dyslipidaemia (dichotomous), and number of antihypertensive drugs (continuous).

Boldface indicates statistical significance (p<0.05).

**Table s9:** Mean differences in HR^a^, SBP^b^ and ^c^nocturnal SBP fall between quintiles of MEDLIFE index (n=2,148).

|  | **Q1** | **Q2** | **Q3** | **Q4** | **Q5** | **p for trend** |
| --- | --- | --- | --- | --- | --- | --- |
| **MEDLIFE index,** score range | 6-12 | 13-14 | 15 | 16-17 | 18-25 |  |
| ***Daytime* HR,** bpm | | | | | | |
| Model 3 | 1 (Ref.) | -0.22 (-1.29, 0.86) | -0.70 (-1.98, 0.57) | **-1.87 (-3.00, -0.74)** | **-2.06 (-3.39, -0.73)** | **<0.001** |
| ***Nighttime* HR,** bpm | | | | | | |
| Model 3 | 1 (Ref.) | -0.82 (-1.78, 0.14) | -0.94 (-2.07, 0.20) | **-2.17 (-3.18, -1.17)** | **-2.31 (-3.49, -1.12)** | **<0.001** |
| ***24-hour* HR,** bpm | | | | | | |
| Model 3 | 1 (Ref.) | -0.36 (-1.35, 0.64) | -0.70 (-1.88, 0.48) | **-1.86 (-2.91, -0.81)** | **-1.93 (-3.16, -0.69)** | **<0.001** |
| ***Nighttime* SBP,** mmHg | | | | | | |
| Model 4 | 1 (Ref.) | -0.66 (-1.87, 0.56) | 0.23 (-1.20, 1.67) | -0.43 (-1.71, 0.85) | **-2.26 (-3.76, -0.76)** | **0.038** |
| ***Nocturnal* SBP *fall*, %** | | | | | | |
| Model 3 | 1 (Ref.) | 0.45 (-0.49, 1.39) | -0.21 (-1.32, 0.90) | 0.23 (-0.75, 1.22) | **1.61 (0.45, 2.77)** | 0.065 |

^a^ HR: Daytime, nighttime and 24-hour heart rate. ^b^ SBP: Nighttime systolic blood pressure. ^c^Nocturnal SBP fall: calculated as: ((daytime SBP - nighttime SBP)/daytime SBP) * 100.

Abbreviations: HR: heart rate; ref.: reference; SBP: systolic blood pressure. Model 3: adjusted for sex (dichotomous), age (continuous) and educational level (categorical), smoking status (categorical), body mass index (categorical), total energy consumption (continuous), prevalent cardiovascular disease (dichotomous), prevalent diabetes (dichotomous), prevalent dyslipidaemia (dichotomous), number of antihypertensive drugs (continuous) and 24-hour systolic blood pressure (continuous). Model 4: adjusted for sex (dichotomous), age (continuous) and educational level (categorical), smoking status (categorical), body mass index (categorical), total energy consumption (continuous), prevalent cardiovascular disease (dichotomous), prevalent diabetes (dichotomous), prevalent dyslipidaemia (dichotomous), number of antihypertensive drugs (continuous), and daytime systolic blood pressure (continuous).

Boldface indicates statistical significance (p<0.05).
